# Bottlebrush polymer conjugates for enhanced antisense oligonucleotide therapy in myotonic dystrophy type 1

**DOI:** 10.1093/nar/gkag619

**Published:** 2026-06-24

**Authors:** Yao Li, Christopher Oetheimer, Yuyan Wang, Gyu Seong Heo, Jiaqi Wu, Rong Chang, Wei Zhang, Elle Schneider, Junjie Chen, Yang Fang, Yun Wei, Keqing Nian, Hengli Zhang, Lauren Sherman, Yongjian Liu, Ke Zhang

**Affiliations:** Department of Bioengineering, Northeastern University, Boston, MA 02115, United States; pacDNA Inc., Natick, MA 01760, United States; Department of Chemistry and Chemical Biology, Northeastern University, Boston, MA 02115, United States; Department of Bioengineering, Northeastern University, Boston, MA 02115, United States; Department of Radiology, Washington University School of Medicine, Saint Louis, MO 63110, United States; Laboratory of Single-Cell Genomics and Population Dynamics, The Rockefeller University, New York, NY 10065, United States; Department of Chemistry and Chemical Biology, Northeastern University, Boston, MA 02115, United States; Department of Bioengineering, Northeastern University, Boston, MA 02115, United States; Department of Biology, Northeastern University, Boston, MA 02115, United States; College of Professional Studies, Northeastern University, Boston, MA 02115, United States; pacDNA Inc., Natick, MA 01760, United States; Department of Chemistry and Chemical Biology, Northeastern University, Boston, MA 02115, United States; Department of Chemistry and Chemical Biology, Northeastern University, Boston, MA 02115, United States; Department of Bioengineering, Northeastern University, Boston, MA 02115, United States; Department of Bioengineering, Northeastern University, Boston, MA 02115, United States; Department of Biology, Northeastern University, Boston, MA 02115, United States; Department of Radiology, Washington University School of Medicine, Saint Louis, MO 63110, United States; Department of Bioengineering, Northeastern University, Boston, MA 02115, United States; Department of Chemistry and Chemical Biology, Northeastern University, Boston, MA 02115, United States; Department of Chemical Engineering, Northeastern University, Boston, MA 02115, United States

## Abstract

Oligonucleotides are a promising class of genetic medicine for myotonic dystrophy type 1 (DM1), the most common adult-onset muscular dystrophy. However, poor muscle distribution of nucleic acid drugs following systemic administration has hindered drug development, and no curative treatment currently exists. DM1 pathology requires drug localization to the nucleus, where pathogenic mutant RNA is sequestered, posing additional challenges after cellular internalization regarding endosomal escape and nuclear uptake. Here, we show that a locked nucleic acid oligonucleotide targeting mutant CUG repeat RNA tracts, conjugated to a bottlebrush polymer, exhibits improved muscle distribution and potent correction of DM1-associated splicing dysregulation in a DM1 mouse model. Significant improvements in myotonia, body weight, and grip strength were observed. The conjugate was well tolerated following 12 weeks of weekly intravenous administration. These findings suggest that bottlebrush polymer conjugates may overcome key limitations of conventional oligonucleotide therapeutics for neuromuscular conditions, with potential to become a potent and cost-effective DM1 therapy.

## Graphical Abstract

**Graphical Abstract:**
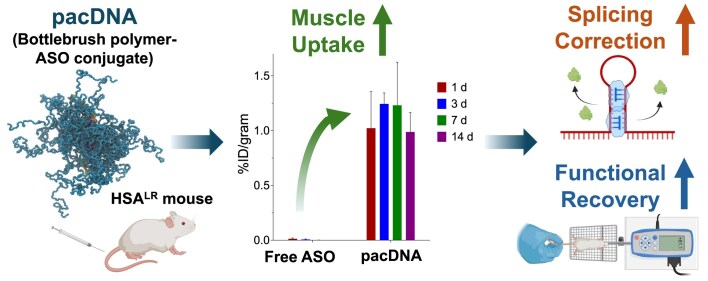

## Introduction

Myotonic dystrophy type 1 (DM1) is a multisystem muscle-wasting disease caused by an unstable CTG repeat expansion mutation in the 3′-untranslated region (UTR) of the DM1 protein kinase (DMPK) gene. DM1 affects 1 in 2100 individuals and is the most common form of adult-onset muscular dystrophy [1]. The autosomal dominant mutation lengthens in successive generations, leading to increased disease severity and earlier symptom onset [2]. Pathogenesis is primarily attributed to the loss of function of muscleblind-like (MBNL) RNA-binding proteins, key regulators of pre-mRNA splicing. MBNL proteins colocalize with DMPK CUG-repeat (CUG^exp^) RNA hairpins, forming large nuclear aggregates or “foci,” leading to altered signaling pathways and gene splicing abnormalities [3]. The HSALR20b (HSA^LR^) mouse model, which carries a (CTG)_220_ repeat in the 3′-UTR of a human skeletal muscle actin transgene, recapitulates the RNA gain-of-function pathology of DM1 in skeletal muscle with high fidelity and reproducibility, making it well-suited for evaluating muscle-targeted therapeutics [4].

Antisense oligonucleotides (ASOs) are a growing class of drugs with attractive qualities, including high specificity, customizability, and robust manufacturing. ASOs exert their therapeutic effect by hybridizing to and blocking or enzymatically degrading target RNA. ASOs have gained considerable recent commercial successes, as seen in multiple exon-skipping therapeutics for Duchenne muscular dystrophy (DMD) and a groundbreaking spinal muscular atrophy drug, highlighting the promise of ASOs for neuromuscular diseases [5, 6]. In DM1, targeting CUG^exp^ RNA can prevent secondary structure formation and limit entrapment of MBNL proteins, leading to recovery in the pathogenic alternative splicing responsible for the DM1 phenotype [3, 7–9]. Interestingly, in preclinical studies, RNase H-inactive steric-blocking ASOs have been reported to achieve significant repeat RNA reduction accompanied by partial splicing correction [8, 10–12]. While the precise mechanism has not been identified, one possibility is that the destabilization of the RNA–MBNL protein complex by the competing ASO leads to enhanced nuclear export of repeat RNA and degradation [11].

Traditional ASO efficacy in muscle following systemic administration is limited by rapid renal clearance, low membrane permeability, and poor biodistribution to target tissues [13–16]. Various chemical modifications, including 2′ ribose modifications, internucleotide linkages, and backbone replacements, have been developed to increase nuclease resistance, elevate duplex stability, and improve tropism to targeted tissues [17, 18]. Several delivery strategies have also been employed to increase ASO muscle biodistribution, cellular uptake, endosomal escape, and nuclear trafficking, including conjugation with cationic cell-penetrating peptides and transferrin-receptor 1 (TfR1) targeting antibodies [12, 19, 20]. While these approaches show potential in clinical studies, there remains room for improvement in splicing correction efficiency, innate/adaptive immune responses, and toxicity [21–26]. To date, no approved curative treatment exists for DM1, with current therapies focusing on symptom management [27].

We have previously shown that conjugation of oligonucleotides to branched polyethylene glycol (PEG) bottlebrush polymers can lend entropic shielding to the oligonucleotides without interfering with on-target hybridization, thereby limiting off-target interactions and immunogenicity [28–32]. While structurally simple, these conjugates, termed pacDNA (polymer-augmented conjugates of DNA), evade renal clearance due to their large size (∼300 kDa, ∼30 nm), improve plasma pharmacokinetics (25-fold and 19-fold increase in elimination half-life and area under the curve, respectively, versus free oligonucleotide) [28], and enhance biodistribution to a variety of organs and tissues through the multiple-pass effect and improved membrane adsorption [33–37]. Earlier studies demonstrate that pacDNA, owing to its near-neutral surface, is taken up predominantly via mixed endocytosis/pinocytosis pathways and trafficked through endolysosomal compartments [28, 38, 39]. As demonstrated by the activity of both noncleavable and bioreducible versions of pacDNA, productive delivery likely does not require linker cleavage prior to target engagement [28, 33, 37]. The results of this study in DM1 mice further suggest nuclear localization of the pacDNA, though the precise efficiency of nuclear delivery remains to be defined. Here, we report the results of a proof-of-concept study testing a pacDNA conjugate targeting toxic CUG^exp^ RNA for the treatment of DM1.

## Materials and methods

### Experimental design

This study evaluated the delivery efficiency, therapeutic efficacy, and safety of bottlebrush polymer conjugated locked nucleic acid (LNA) ASOs (pacDNA) for the treatment of DM1. The HSA^LR^ transgenic mouse model was selected for its well-characterized DM1-like features, including CUG^exp^ RNA nuclear foci, splicing dysregulation, myotonia, and muscle weakness.

To assess the delivery performance of pacDNA, we first examined its biodistribution in skeletal muscle of treated mice and its nuclear localization in DM1 patient-derived cells, to confirm both tissue uptake and intracellular target engagement. To evaluate dose-dependent efficacy, HSA^LR^ mice were injected intravenously with pacDNA at 5.3, 21.2, or 42.4 mg/kg. To assess durability and long-term tolerability, a separate cohort received weekly intravenous injections for 12 consecutive weeks, beginning at 8 weeks of age.

Therapeutic outcomes were assessed by RNA sequencing to quantify transcriptome-wide splicing correction and gene expression changes. Functional improvement was evaluated by pinch-induced myotonia scoring, grip strength testing, and body weight monitoring. Safety was evaluated through cytokine and chemokine profiling, anti-PEG IgM/IgG quantification, and serum biomarkers of hepatic and renal function.

### Materials and reagents

Methoxy PEG Amine, HCl Salt (A3052) was purchased from Jenkem Technology, USA. Phosphoramidites and supplies for DNA synthesis were obtained from Glen Research Co., USA. All other materials were obtained from MilliporeSigma, Fisher Scientific Inc., USA, or VWR International LLC, USA, and were used as received unless otherwise indicated.

### Instrumentation

Reversed-phase high-performance liquid chromatography (RP-HPLC) was performed on a Waters (Waters Co., MA, USA) Breeze 2 HPLC system coupled to a Symmetry® C18 3.5 μm, 4.6 × 75 mm reversed-phase column and a 2998 PDA detector, using TEAA buffer (0.1 M) and HPLC-grade acetonitrile as mobile phases. Aqueous GPC (gel permeation chromatography) measurements were performed on a Waters Breeze 2 GPC system equipped with Ultrahydrogel™ 1000, Ultrahydrogel™ 500, and Ultrahydrogel™ 250, 7.8 Å × 300 mm column, and a 2998 PDA detector (Waters Co., MA, USA). 0.1 M sodium nitrate (pH 7.0) was used as the eluent, running at a flow rate of 0.8 ml/min.

*NN*-dimethylformamide (DMF) GPC was performed on a TOSOH EcoSEC HLC-8320 GPC system (Tokyo, Japan) equipped with a TSKGel GMHHR-H, 7.8 × 300 mm column, and RI/UV-Vis detectors. HPLC-grade DMF with 0.05 M LiBr was used as the mobile phase, with samples run at a 0.5 ml/min flow rate. GPC calibration was performed with a PEG/polyethylene oxide Readycal set (Sigma–Aldrich, USA).

### Cell culture and animals

Human DM1 patient-derived fibroblasts carrying an ∼2000 CTG repeat mutation in the DMPK gene and healthy control fibroblasts were obtained from the NIGMS Human Genetic Cell Repository at the Coriell Institute for Medical Research (GM03989, GM07492). Cells were passaged and cultured in Eagle’s minimum essential medium with 10% fetal bovine serum (FBS), 1% antibiotic antimycotic, and 1% nonessential amino acid solution in 5% CO_2_ at 37°C. All cell counting was performed using Trypan Blue and a hemocytometer. Cells were cryopreserved for storage in 90% FBS with 10% dimethyl sulfoxide (DMSO) (1 ml).

Homozygous HSA^LR^ transgenic mice (human skeletal actin long repeat line 20b, Strain #032031) and FVB/N (Strain #001800) were purchased from The Jackson Laboratory, USA [4]. Animal experiments were conducted at Northeastern University and carried out in accordance with approved Institutional Animal Care and Use Committee guidelines (protocol number: 22-0309). Adult mice aged ∼8 weeks were injected via tail vein with pacDNA, free ASO, or scramble/brush polymer controls dissolved in 200 µl of sterile phosphate-buffered saline (PBS). Animals were humanely euthanized by CO_2_ at indicated time points for tissue collection. Blood for serum analysis assays was collected from the submandibular vein at indicated time points.

### Antisense oligonucleotides

Custom oligonucleotides were synthesized on a Dr. Oligo 48 DNA synthesizer (Biolytic Lab Performance, Inc., Fremont, CA). Short-length single-stranded DNA with a sequence of 5′-CAGCAGCAG-3′ of LNA-modified bases was made, and dT-DBCO was attached at the 5′ end for downstream “click” reactions. All-LNA scramble control oligonucleotides (pacDNA-Scr, 5′-CGCACAAGG-3′) were synthesized in parallel. A fluorescent probe with a sequence of 5′-(Cy-3)-C**A**GC**A**GC**A**GCAG**C**AG**C**AG**C**A-3′ (bold = LNA modification) was synthesized for fluorescence *in situ* hybridization (FISH) assays [40]. All DNA was synthesized using 1 μmol controlled pore glass (CPG) dT columns or 200 nmol Cy-3 CPG columns. Oligonucleotides were cleaved from the CPG solid support and deprotected in aqueous ammonium hydroxide solution (28%–30% NH_3_) at room temperature for 16 h. Free oligonucleotides were purified by RP-HPLC. If necessary, dimethoxytrityl protecting groups were removed by treatment with 20% acetic acid for 1 h followed by three ethyl acetate extractions. Purified DNA was quantified on a NanoDrop® ND-1000 UV-Vis Spectrophotometer and lyophilized for storage at −20°C.

### Synthesis and purification of pacDNA conjugates

Norbornenyl bromide was synthesized using a two-step synthesis involving maleimide (one equiv.), furan (one equiv.), 1,4-dibromobutane (four equiv.), and K_2_CO_3_ (five equiv.) as previously described [28]. 5-Norbornene-2-acetic acid succinimidyl ester (norbornenyl NHS ester) was synthesized via a reaction of exo-5-norbornene carboxylic acid (MilliporeSigma, 1 equiv.) and N-hydroxysuccinimide (Thermo Fisher, 1.4 equiv.) in the presence of 1-ethyl-3-(3-dimethylaminopropyl)carbodiimide (EDCI) (MilliporeSigma, 1.3 equiv.) as a carboxyl-activating agent in dichloromethane (DCM) for 16 h at room temperature. The product was purified by silica column chromatography with a 2:1 hexane:ethyl acetate mobile phase. Norbornene-PEG was synthesized as previously described by reacting norbornenyl NHS ester (1.5 equiv.) and methoxy-PEG amine HCl salt (JenKem Technology USA, 1 equiv.) in the presence of *N,N*-diisopropylethylamine (DIPEA) (Thermo Fisher, 1.5 equiv.) in DCM for 16 h at room temperature [41]. The product was precipitated in cold anhydrous ethyl ether, washed 3×, and lyophilized for storage at −20°C. Third-generation Grubbs’ catalyst was prepared by reacting 2nd Generation Grubbs Catalyst (MilliporeSigma, 1 equiv.) with 3-bromopyridine (MilliporeSigma, 100 equiv.), followed by precipitation in cold hexane as previously described [42].

Diblock bottlebrush polymer was synthesized via ring-opening polymerization of norbornenyl bromide and norbornenyl PEG using 3rd generation Grubbs’ catalyst [28]. DMF-GPC was performed to quantify the molecular weight, polydispersity index, and yield of the bottlebrush polymer. Purified bottlebrush polymer was conjugated with an azide group by substitution reaction with sodium azide (Thermo Fisher, 100 equiv.). Azide-functionalized bottlebrush polymer (1 equiv.) and DBCO (dibenzocyclooctyne)-modified DNA (2.2 equiv.) were reacted in a standard copper-free “click chemistry” reaction for 24 h at 55°C as previously described [43]. The conjugated pacDNA product was purified by aqueous GPC with a 1 M sodium nitrate mobile phase in an Ultrahydrogel 250 Å column. ASO loading per polymer was assessed by aqueous GPC, comparing the intensities of free ASO and bottlebrush polymer conjugated peaks. Average conjugation efficiency is ∼90%, yielding 2.0 ASOs loaded per polymer. Gel electrophoresis was performed using 2% agarose gel in 1× tris/borate/EDTA (TBE) buffer with a running voltage of 120 V. Gel images were acquired on an Alpha Innotech Fluorochem Q imager. DLS and ζ potential data were recorded on a Malvern Zetasizer Nano-ZSP (Malvern, UK). After DNA conjugation and purification, the pacDNA was desalted using a NAP-25 column (Cytiva) and lyophilized for storage at −20°C.

### Fluorescence *in situ* hybridization and confocal microscopy

Human fibroblasts were seeded in a 12-well confocal plate and treated with various concentrations of pacDNA or controls for 24 h. Then, cells were fixed in 4% PFA at 4°C followed by permeabilization with 70% ethanol overnight. Pre-hybridization was then performed using a buffer (30% formamide, 2× SSC) for 10 min. Hybridization was performed using a FISH probe 5′-(Cy-3)-C**A**GC**A**GC**A**GCAG**C**AG**C**AG**C**A-3′ (bold = LNA modified, 2 ng/µl) in a buffer containing formamide, SSC, bovine serum albumin, yeast tRNA, dextran sulfate, and vanadyl ribonucleoside complex [40]. Cells were stained with DAPI (Thermo Fisher) for 10 min and washed 3× with PBS. Images were acquired on a Zeiss LSM 880 confocal laser scanning microscope under identical settings (Carl Zeiss Ltd., Cambridge, UK). Foci were counted by eye in ∼250 nuclei in each sample group. Confocal imaging of live cells was conducted by a similar procedure, omitting the steps of fixation, permeabilization, and hybridization. Dual-labeled pacDNA was synthesized using Cy5-L9-DBCO ASO, which was then conjugated to Cy3-labeled bottlebrush polymer at a 1:1 Cy3:Cy5 ratio for qualitative assessment of signal colocalization *in vitro* as previously described [33].

### RNA isolation

HSA^LR^ or FVB/N wild-type (WT) mice were euthanized, and muscle samples were collected from the quadriceps and flash frozen in liquid nitrogen and stored at −80°C. Individual sections were homogenized in a lysis buffer containing 1% β-mercaptoethanol (BME) on a Beadblaster 24R Microtube Homogenizer (Benchmark Scientific, NJ, USA). Total cellular RNA was extracted using the RNeasy® Fibrous Tissue Mini Kit (Qiagen), and RNA quality and quantity were assessed on a NanoDrop® ND-1000 UV-Vis Spectrophotometer.

### ^89^Zr radiolabeling to measure biodistribution

For radiolabeling, pacDNA was synthesized with two desferrioxamine (siderophore-derived chelator) functionalities per molecule, which were used to chelate ^89^Zr-oxalate at a ratio of ~500 kBq to 1 μg. The radiochemical labeling yields were monitored by instant radio-thin-layer chromatography. Radio-fast protein liquid chromatography with a size-exclusion column was used to remove unchelated ^89^Zr. To evaluate biodistribution/clearance and perform small animal PET (positron-emission tomography) imaging, CD-1 mice were i.v. injected with ~500 MBq of pacDNA in 100 μl saline. Mice were anesthetized with inhaled isoflurane and re-anesthetized before euthanasia by cervical dislocation at predetermined time points (24 h, 3, 7, and 14 days post-injection, *n* = 4/time point). Organs/tissues of interest were collected, weighed, and radio-counted. Standards were prepared and measured along with the samples to calculate the percentage of the injected dose per gram of tissue (%ID/g). PET scans were carried out at two of the four time points (24 h and 7 days) prior to the euthanization of the mice. The microPET images were corrected for attenuation, scatter, normalization, and camera dead time and co-registered with microCT images.

### RNA sequencing

Isolated RNA from quadriceps was sent for total RNA sequencing (Novogene), targeting 40 million reads per sample. After polyA enrichment and quality control, libraries were sequenced on an Illumina PE150 system to obtain paired-end reads of 150 bp. After trimming and alignment, reads were mapped to the mouse genome (*Mus musculus* GRCm39), and analysis was performed in RStudio using DESeq2, clusterProfiler, fgsea, gsva, and Rsubread packages. The Benjamini–Hochberg method was applied to account for multiple testing. Adjusted *P*-values (padj <.05) were then used to filter differentially expressed genes. Gene set enrichment analysis (GSEA) and gene set variation analysis (GSVA) were performed to assess functional gene expression changes after pacDNA-L9 treatment. To identify genes showing recovery patterns after treatment, we calculated recovery scores for each differentially expressed gene between WT and NT conditions. The Recovery Score was defined as the ratio of the absolute difference between the normalized gene expression (*E*) in the treated cohort and WT to the sum of absolute differences between the treated cohort and WT, and the treated cohort and NT. Lower scores indicate better recovery toward the WT state. The top 25% of genes with the lowest recovery scores were selected for heatmap visualization. Gene expression values were normalized using DESeq2’s size factors and transformed into *z*-scores for heatmap visualization.


\begin{eqnarray*}\textrm{Recovery}\ \textrm{Score} =\frac{\left| E_T - E_{\mathrm{WT}} \right|}{\left[ \left| E_T - E_{\mathrm{WT}} \right| + \left| E_T - E_{\mathrm{NT}} \right| \right]}.\end{eqnarray*}

Levels of endogenous genes containing short (CUG)_*n*_ (*n* = 2–25) RNA tracts were examined for potential off-target effects of the pacDNA (Supplementary Table S3). Human ACTA1 transgene expression was quantified from sequencing data by aligning fastq files to the reference sequence (Gene ID: 58) using HISAT2 (v2.2.1) [44]. The resulting SAM files were converted to sorted BAM files with SAMtools (v1.13), and read counts mapping to hACTA1 were normalized to GTF2B expression as previously described [45].

Splicing events were calculated using the replicate multivariate analysis of transcript splicing statistical model across five distinct splicing modes (SE, A5SS, A3SS, MXE, RI) [46]. Events were considered significant if they met both an FDR threshold (<0.01) and a minimum of 10 junction counts per sample. Splicing mode analysis was performed by calculating the fraction of disease-associated splicing events (FDR < 0.01 in NT versus WT) that were normalized by treatment, defined as events no longer reaching significance (FDR > 0.01) in the pacDNA versus WT comparison.

The percent corrected PSI for each individual splicing event *x* was calculated using the following formula, where PSI_WT_(*x*), PSI_NT_(*x*), and PSI_T_(*x*) represent the average PSI values for splicing event *x* in the WT, nontreated, and treated cohorts, respectively. The percent corrected PSI for each treatment group was calculated as the mean of the percent corrected PSI values across the 21 DM1-associated splicing events for each animal.


$$\\mathrm{Percent\ Corrected\ PSI}_x(\%) =\frac{\mathrm{PSI}_{NT}(x)-\mathrm{PSI}_{T}(x)}{\mathrm{PSI}_{NT}(x)-\mathrm{PSI}_{WT}(x)}\times 100$$

### Behavioral evaluation of myotonia and grip strength in HSA^LR^ mice

#### Tail-pinch-evoked myotonia scoring assay

To evaluate skeletal muscle myotonia, a blinded behavioral assay was conducted based on a series of tail-pinches. Mice were gently pinched on the lower back, ~1 cm cranial to the base of the tail, 15 times per session using blunt forceps. A semi-quantitative scoring system was applied to assess the severity of delayed hindlimb relaxation following each pinch, defined as follows:

- 3 = severe myotonia (bilateral hindlimb extension lasting >1 s)
- 2 = moderate myotonia (0.5–1 s)
- 1 = mild myotonia with quick recovery (<0.5 s)
- 0.5 = unilateral hindlimb extension
- 0 = no myotonic response

Mice were tested in a randomized order to avoid handling bias, and a 20-min rest was provided after every eight pinches to minimize fatigue. A mean score was calculated from the 15 trials to represent the myotonia severity of each individual mouse.

#### Grip strength and body weight measurements

Forelimb and hindlimb grip strength was measured using a Grip Strength Meter (Bioseb) at baseline and selected time points throughout the treatment period. Four independent readings were taken per mouse per session, and the average value was recorded. Results were compared with age- and sex-matched untreated HSA^LR^ and FVB/N WT control mice. Body weight was measured weekly using a calibrated digital scale.

#### Anti-PEG immune response

HSA^LR^ mice were administered PBS or pacDNA-L9 (10.6 mg/kg) weekly. As a positive control for PEG immunogenicity, a separate group of HSA^LR^ mice received 4 mg/kg of PEG-keyhole limpet hemocyanin (PEG-KLH), a known immunogenic PEG conjugate. Plasma (100 µl) was collected 1 and 3 weeks after the final dose. Anti-PEG IgG and IgM concentrations were measured using ELISA kits (Cat #PEGG-1 and #PEGM-1, Life Diagnostics, Inc.) according to the manufacturer’s protocol.

#### Multiplex analysis of cytokines and blood markers

HSA^LR^ mice were treated with pacDNA-L9, free L9 ASO, PBS, or lipopolysaccharide (LPS, 2 mg/kg) positive control. Plasma was collected 4 h after the final injection, and multiplex analysis of cytokines was performed using the Luminex™ 200 system (Luminex, Austin, TX, USA) by Eve Technologies Corp. (Calgary, Alberta). The 32-plex panel included Eotaxin, GM-CSF, IL-1α, IL-1β, IL-2, IL-3, IL-4, IL-5, IL-6, IL-10, IL-12(p70), IL-13, IL-15, KC, LIX, MCP-1, and TNFα. The assay sensitivities of these markers range from 0.3–30.6 pg/ml. Individual analyte sensitivity values are available in the MilliporeSigma MILLIPLEX® MAP protocol. Serum chemistry biomarkers (Albumin, ALT, AST, ALP, blood urea nitrogen, calcium, creatinine kinase, creatinine, phosphorus, total protein, and total bilirubin) were quantified by Idexx Bioanalytics (Columbia, MO) according to company protocols.

#### Statistical analysis

For two-group and multigroup comparisons, we used unpaired two-tailed *t*-tests or multigroup analysis of variance (ANOVA) (GraphPad Prism 10.4.1 software, GraphPad, Inc.). We used the *F*-test to compare variances between the two groups. *P*-values denoted by * symbols were calculated in Prism. Data are presented as the mean ± SEM or mean ± s.d., with *n* values noted in all figure captions. A two-tailed nonparametric *t*-test was applied to compare two groups that have a statistically significant difference in variance (Foci counting, myotonia, body weight). One-way ANOVA followed by Tukey’s multiple comparison test was applied for percent corrected PSI analysis of the DM1 splicing panel and grip strength functional study.

## Results

### pacDNA enables the targeting of CUG^exp^ nuclear foci

**Figure 1.**
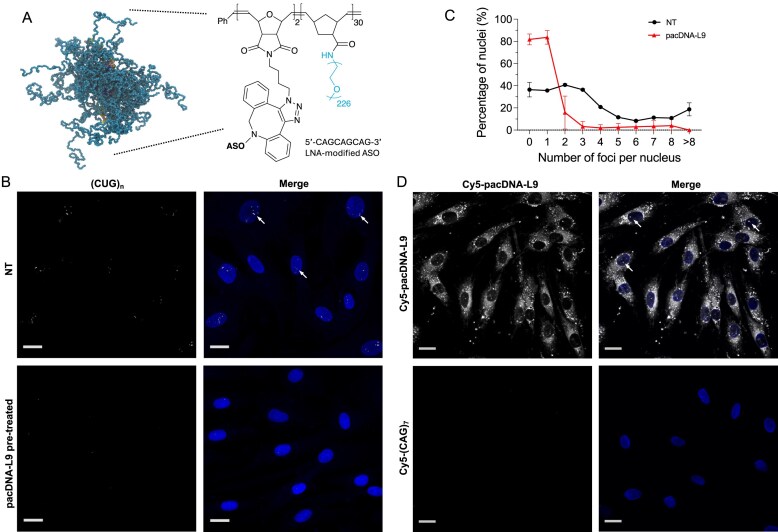
pacDNA-L9 enables targeting of CUGexp nuclear foci in primary human DM1 fibroblasts. (**A**) Chemical structure and molecular dynamics simulation of the pacDNA in water. (**B**) FISH of primary human DM1 fibroblasts (GM03989, 2000 CTG repeats) showing CUG^exp^ foci signal (highlighted with arrows) and DAPI-stained nuclei (upper two panels). FISH images of GM03989 cells following treatment with 1 μM pacDNA-L9 for 24 h (lower two panels). (**C**) Quantification of # of foci per nucleus in nontreated and pacDNA-L9-treated cells (250 nuclei counted per sample). (**D**) GM03989 cells treated with 1 μM Cy5-pacDNA-L9 for 24 h. Engagement with nuclear foci highlighted by arrows (upper two panels). GM03989 cells treated with 1 μM free Cy5-(CAG)_7_ for 24 h (lower two panels). Scale bars: 20 μm.

Bottlebrush polymers were synthesized by sequential ring-opening metathesis polymerization of 7-oxanorbornenyl bromide (ONBr) and norbornenyl PEG_10 kDa_ (NPEG) to yield diblock polymers (pONBr_5_-*b*-pNPEG_30_), followed by azide substitution of the bromide as previously described (Fig. 1A and Supplementary Fig. S1A) [28]. An LNA oligonucleotide complementary to the CUG^exp^ repeat (5′-CAGCAGCAG-3′, fully LNA-modified, phosphodiester linkages, termed L9) with a DBCO functionalized 5′ end was conjugated to the polymer backbone via the strained cyclooctyne copper-free click reaction (∼2.0 ASO per polymer) [43]. Gel electrophoresis, dynamic light scattering, and aqueous GPC confirmed that the pacDNA-L9 particles are uniform in size, free from aggregation, and possess a near-neutral surface charge (zeta potential ζ ∼ −2.9 mV compared to ζ ∼ −38.0 mV for the free ASO) (Supplementary Fig. S1B–D) [28, 30].

FISH was performed in primary human DM1 fibroblasts carrying a (CTG)_2000_ repeat mutation in the DMPK gene (GM03989, Coriell Institute for Medical Research). The CUG^exp^ foci characteristic of DM1 were detected as punctate patterns within the cell nuclei (Fig. 1A). When the cells were pre-treated with 1 μM pacDNA-L9 for 24 h, the number of foci detected by FISH per nucleus decreased (Fig. 1B), suggesting that the pacDNA-L9 conjugate or L9 fragments generated by intracellular nuclease activity can enter the nucleus and engage with target CUG^exp^ RNA without a transfection agent. Quantitatively, the percentage of nuclei with no detected foci increased from 16.8% to 44.7%, with all counted nuclei in the treated group possessing three or fewer foci (Fig. 1C). Additional replicate FISH experiments using escalating concentrations of pacDNA-L9 are provided in Supplementary Fig. S2.

When live cells were treated with fluorescently labeled Cy5-pacDNA-L9 or free Cy5-(CAG)_7_ for 24 h and imaged, the conjugate displayed improved cell and nuclear uptake relative to free ASO. The pacDNA showed nuclear accumulation in punctate patterns, suggesting its ability to engage with pathogenic target foci, while the free ASO showed little to no uptake by the cell (Fig. 1D). Using a transfection agent (Lipofectamine 3000) with free ASO significantly improves cellular uptake and also allows nuclear foci to be detected (Supplementary Fig. S3). Images collected from 24 to 96 h show no obvious change in ASO localization or nuclear distribution (Supplementary Fig. S4). To further explore the intracellular stability of pacDNA-L9, we conducted a dual-labeling study, in which Cy5-L9-DBCO was conjugated to Cy3-labeled brush polymer as previously described [33]. After 24 h incubation with GM03989 cells, signals remained largely colocalized, with both components accumulating in endosomes and to a lesser degree in the nuclei of cells (Supplementary Fig. S5). These results suggest that pacDNA-L9 can engage with nuclear targets as an intact structure despite its large size.

### pacDNA enhances pharmacokinetics and biodistribution to skeletal and cardiac muscles

**Figure 2.**
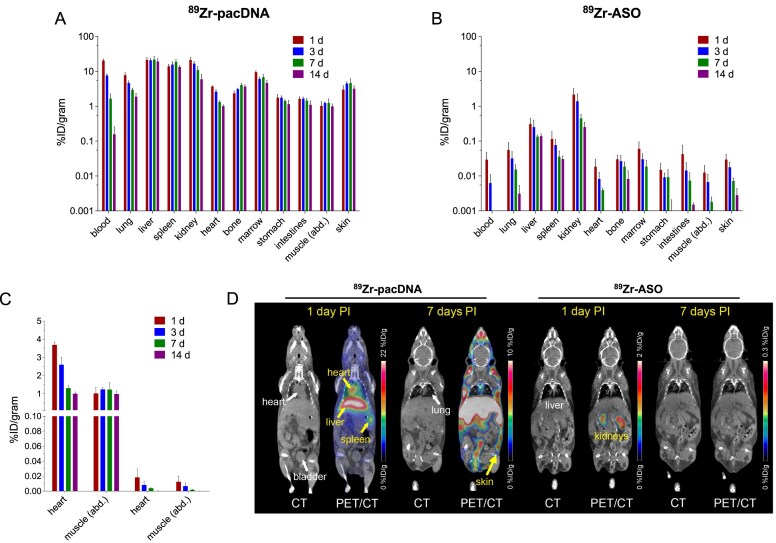
Biodistribution of radiolabeled pacDNA and free ASO. (**A, B**) *Ex vivo* biodistribution of ^89^Zr-labeled pacDNA and ^89^Zr-ASO in various tissues and organs measured over 14 days (*n* = 4/time point) in CD-1 mice. The *y-*axis shows the percent of injected dose per gram of tissue (%ID/g). (**C**) %ID/g comparison of heart and muscle of ^89^Zr-pacDNA (left) and ^89^Zr-ASO injected mice (right). (**D**) PET scans of ^89^Zr-pacDNA (left) and ^89^Zr-ASO injected mice (right) at 1 and 7 days post-injection. Scale bars represent %ID/g. Error bars indicate ± s.d.

To evaluate the biodistribution profile of pacDNA, a 14-day longitudinal biodistribution study was performed using CD-1 mice and ^89^Zr-radiolabeled pacDNA or unconjugated ^89^Zr-ASO bearing a poly(T) sequence. Of note, this sequence is entirely phosphodiester-linked and does not represent the pharmacokinetic improvements afforded by modification chemistries such as phosphorothioate (PS) oligonucleotides [47, 48]. The probe oligonucleotide was synthesized with two desferrioxamine functionalities per molecule, which were used to chelate ^89^Zr-oxalate. Radionuclide offers better real-time, quantitative, and deep-tissue imaging capabilities compared to fluorescence-based techniques. The use of ^89^Zr, which has a relatively long *t*_1/2_ of 3.27 days, makes it possible to monitor the injected dose for up to 2 weeks. *In vitro* studies in mouse serum demonstrated >95% stability after 9 days, ensuring the accurate tracking of radiolabeled conjugates *in vivo*. CD-1 mice were i.v. injected with ~500 MBq of sample in 100 μl saline. At predetermined time points (24 h, 3, 7, and 14 days post-injection, *n* = 4/time point), organs/tissues of interest were collected, weighed, and radio-counted (Fig. 2A and B).

Elevated retention of pacDNA conjugate was observed in blood pool organs (blood, heart, lung) as well as in mononuclear phagocyte system organs (liver, spleen). Moderate uptake was observed for peripheral tissues, including the abdominal and thigh muscles [∼1% injected dose (ID)/g] and skin (∼4% ID/g). Skeletal muscle emerges as one of the highest per-organ accumulation sites for pacDNA, containing 12.7% of injected dose at 72 h (only exceeded by the liver at 21.9%). The rapid decrease in heart drug levels from ∼4% ID/g at 1 day may reflect the initially high cardiac exposure driven by both the high perfusion rate and capillary density of heart tissue and the elevated circulating levels of pacDNA immediately following systemic administration [49]. Cardiac delivery may serve as a therapeutic advantage in multisystemic diseases such as DM1, where cardiac pathology is a major contributor to morbidity [50]. Approximately 40% of the injected pacDNA dose was cleared via urine and feces by day 14. Importantly, given that muscle tissues constitute ~40% of the mass of the animal [51], at 1% ID/g, pacDNA enables ∼13% of total injected dose to localize to muscles, representing a 10^2^–10^4^-fold increase over the free oligonucleotide depending on the time point (Fig. 2C). These muscle uptake results also compare favorably with TfR1-targeted antibodies, showing 3- to 10-fold higher muscle concentration [52]. Muscle selectivity, as defined by the ratio of C_max_ in the dose-limiting organ (kidney) versus muscle, also improves significantly (from ∼1680 for free oligonucleotide to 20.4). Notably, the pacDNA conjugate also showed markedly increased accumulation in the gallbladder, pancreas, intestines, stomach, and brain compared with free ASO. Additionally, PET/computed tomography (CT) scans were carried out on day 1 and day 7 before the euthanization of the mice (Fig. 2D). PET/CT imaging shows extended blood retention and significant tissue uptake of the pacDNA, while the free oligonucleotide suffered from rapid renal clearance with little accumulation in non-kidney tissues. A similar biodistribution profile was observed for fluorescently labeled pacDNA-L9 in HSA^LR^ transgenic mice, demonstrating comparable accumulation in skeletal and cardiac muscles (∼1% ID/g) as in CD-1 mice (Supplementary Fig. S6). The level of muscle uptake achieved by pacDNA is favorable compared to targeted approaches using monoclonal antibodies or peptide ligands [12, 52–58]. A comparative analysis of skeletal muscle accumulation across published muscle delivery strategies, expressed as %ID/g to normalize for dose, is provided in Supplementary Fig. S7. Collectively, these data establish that pacDNA is a long-circulating, long-retention vector suitable for systemic delivery to address muscular disorders.

### pacDNA-L9 corrects DM1-associated alternative splicing in HSA^LR^ mice

**Figure 3.**
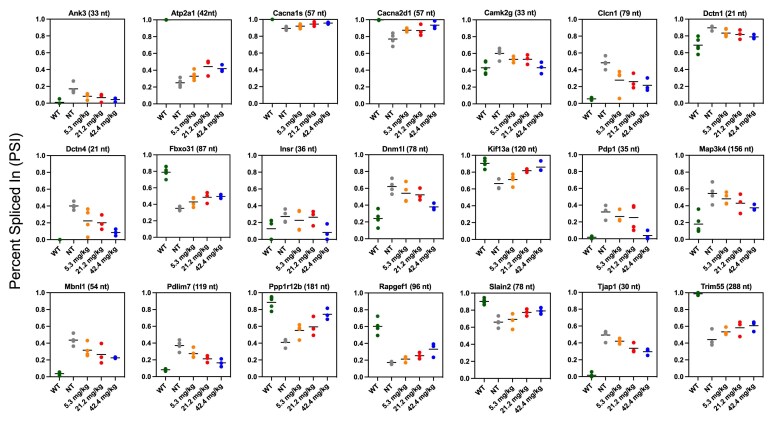
DM1 alternative splicing panel. PSI of 21 DM1-associated splicing events in WT (*n* = 5), nontreated HSA^LR^ (*n* = 4), or pacDNA-L9 5.3 mg/kg (*n* = 4), 21.2 mg/kg (*n* = 3), or 42.4 mg/kg (*n* = 3) treated groups (ASO equivalent). Dosing in terms of full pacDNA-L9 conjugate was 480 mg/kg, 1920 mg/kg (four injections over 2 weeks), and 3840 mg/kg (eight injections over 2 weeks). Mice were euthanized 2 weeks after the final injection.

**Figure 4.**
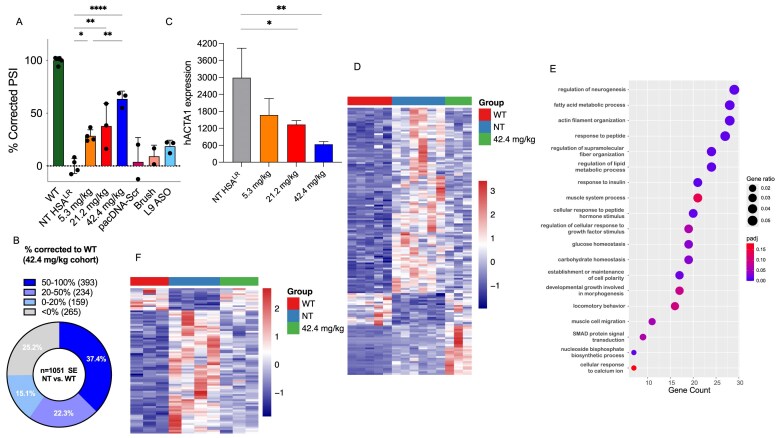
Transcriptomic alterations following pacDNA-L9 treatment. (**A**) Percent corrected PSI across the 21-gene fragment panel (Fig. 3) of DM1-associated splice events in the WT, NT, or pacDNA-L9-treated cohorts (one-way ANOVA with Tukey’s multiple comparison test, ****P < .0001, **P < .01, and *P < .05). Percent values under each group represent the mean percent corrected to WT PSI levels. (**B**) Percent corrected to WT PSI of 1051 significant SE events in nontreated HSA^LR^ mice by 42.4 mg/kg pacDNA-L9 treatment. (**C**) Human ACTA1 transgene expression in quadriceps of nontreated or pacDNA-L9-treated HSA^LR^ mice (expression normalized to GTF2B, mean and ±SEM. of biological replicates, **P < .01, *P < .05, and one-way ANOVA). (**D**) Top 25% most recovered genes following 42.4 mg/kg pacDNA-L9 treatment, visualized by heatmap. (**E**) Biological processes identified by GSEA of genes corrected (padj >.05 versus WT) by 42.4 mg/kg pacDNA-L9 treatment. (**F**) Top 25% most recovered exon bins following 42.4 mg/kg pacDNA-L9 treatment, visualized by heatmap.

HSA^LR^ mice (8 weeks old) were injected i.v. by tail vein with 5.3 mg/kg–42.4 mg/kg (ASO basis; 480–3840 mg/kg full conjugate) of pacDNA-L9, 5.3 mg/kg pacDNA with a scrambled sequence (pacDNA-Scr), 5.3 mg/kg free L9 control, or an equimolar dose of unconjugated bottlebrush polymer (Brush). Total cellular RNA from mouse quadriceps was sequenced 2 weeks post-injection to evaluate gene expression and RNA splicing patterns. Among the many genes affected in DM1 are several encoding membrane ion channel proteins, whose misregulation disrupts normal muscle ion gradients and electrophysiology, leading to hallmark physical manifestations of DM1, such as myotonia, myopathy, and arrhythmia [59–63]. We measured the exon inclusion, or percent spliced-in (PSI), across a panel of 21 alternatively spliced exon events in DM1-relevant genes (Fig. 3) [64–69]. Many of these 21 genes have been identified as direct targets of MBNL splicing proteins and thus serve as robust biomarkers of MBNL protein activity in muscle. Splicing dysregulation improved 2 weeks after pacDNA treatment in a dose-dependent manner. To quantify this effect, the percent-corrected PSI was calculated for each sample, where the mean of nontreated HSA^LR^ mice was normalized to 0 and the mean of WT mice to 100. In pacDNA-L9 treated mice, PSI correction progressively increased with increasing dose, indicating a shift away from DM1-associated splicing patterns and toward WT exon inclusion (Fig. 4A, one-way ANOVA ***P < .01,*P < .05). The 5.3, 21.2, and 42.4 mg/kg pacDNA-L9 cohorts exhibited mean PSI corrections of 28.5%, 37.6%, and 63.4%, respectively, in this 21-gene panel. Notably, several key transcripts associated with specific DM1 phenotypes (e.g., *Clcn1, Mbnl1, Cacna2d1, Cacna1s, Dnm1l*, and *Insr*) [61, 70, 71] displayed strong splicing correction nearly back to WT levels. In contrast, the free bottlebrush polymer and pacDNA-Scr, and control groups displayed low correction near 0% (Supplementary Fig. S8). The free L9 group showed modest correction of 18.7%, consistent with expected LNA ASO exposure in muscle; however this did not reach statistical significance (Supplementary Fig. S9). Considering all skipped exon (SE) events, the highest dose of pacDNA-L9 corrected the PSI of 393 events by 50%–100%, 234 events by 20%–50%, and 159 events by 0%–20% (Fig. 4B). Additionally, treatment with pacDNA-L9 corrected a significant fraction of global alternative splicing. Of 1287 significantly dysregulated splicing events in nontreated HSA^LR^ mice versus WT across five splicing modes (A3SS, A5SS, MXE, RI, SE), 21.2 mg/kg pacDNA-L9 corrected 38%, and 42.4 mg/kg pacDNA-L9 corrected 49% of all events (Supplementary Fig. S10).

Expression of human ACTA1 transgene was quantified in pacDNA-L9-treated mice and nontreated controls, with values normalized to GTF2B expression as previously described [45]. Notably, pacDNA-L9 treatment reduced hACTA1 expression in a dosage-dependent manner, consistent with earlier reports for steric-blocking ASOs, which may be a result of the destabilization of nuclear CUG^exp^ RNA structures leading to their accelerated decay or nuclear export [8, 11]. In the 42.4 mg/kg cohort, hACTA1 expression was reduced by 79% relative to nontreated controls (Fig. 4C).

### pacDNA-L9 shifts the global transcriptome toward WT at both gene expression and splicing levels

To examine the overall effect of pacDNA-L9 treatment at the transcriptomic level, we performed bioinformatic analysis of both gene expression and alternative splicing. From all differentially expressed genes between WT and NT samples (padj <.05, |log2FoldChange| > 1), the top 25% most recovered genes following 42.4 mg/kg pacDNA-L9 treatment were visualized by heatmap. The calculation of a recovery score is described in the “Materials and methods” section. The treatment group exhibited an intermediate recovery pattern, closer to the WT group than to the NT group, suggesting significant restoration of gene expression (Fig. 4D and Supplementary Table S1). Similarly, transcriptomic analysis of exon bins (normalized counts) demonstrated global correction of alternative splicing misregulation following treatment (Fig. 4E and Supplementary Table S2). GSEA of significantly corrected genes (padj >.05 versus WT) revealed functional signatures associated with the restored gene expression (Fig. 4E). Among the top corrected biological processes were fatty acid and lipid metabolic process, glucose homeostasis, and response to insulin, indicating a recovery in metabolism- and energy storage/conversion-related gene expression. Additionally, categories such as muscle system process, actin filament organization, muscle cell migration, and growth factor and morphogenesis suggest restoration of healthy muscle function and integrity [64, 69, 72, 73]. GSVA of the most downregulated gene sets in nontreated HSA^LR^ samples compared to WT further highlights the profound recovery in disease-related gene dysregulation following pacDNA-L9 treatment even in the low-dose cohort (Supplementary Fig. S11A and B).

It has been shown that CUG^exp^ RNA can be cleaved by Dicer into small CUG-repeat RNAs and used as templates for RNA interference (RNAi) of endogenous mRNA transcripts in the cytoplasm, contributing to global alterations of the DM1 transcriptome [72, 74]. Due to the pacDNA’s high muscle concentrations and cell uptake, even the lowest dose (5.3 mg/kg) may be sufficient for mitigating these cytoplasmic RNAi effects. Since the (CAG)_3_ ASO on the pacDNA-L9 has the potential to bind other endogenous (CUG)_n_-containing transcripts, we examined the expression levels of 21 genes with varying (*n* = 2–25) CTG repeats [10, 11, 75]. Tcf4 was slightly downregulated (log_2_fc = −0.36, padj = .03 versus NT). The rest of these genes were unaffected by pacDNA-L9 treatment at the three tested doses, confirming the absence of off-target modulation of short (CUG)_*n*_-containing transcripts across the dose range tested (Supplementary Fig. S11C and Supplementary Table S3).

### pacDNA-L9 alleviates myotonia and improves muscle strength in HSA^LR^ mice

**Figure 5.**
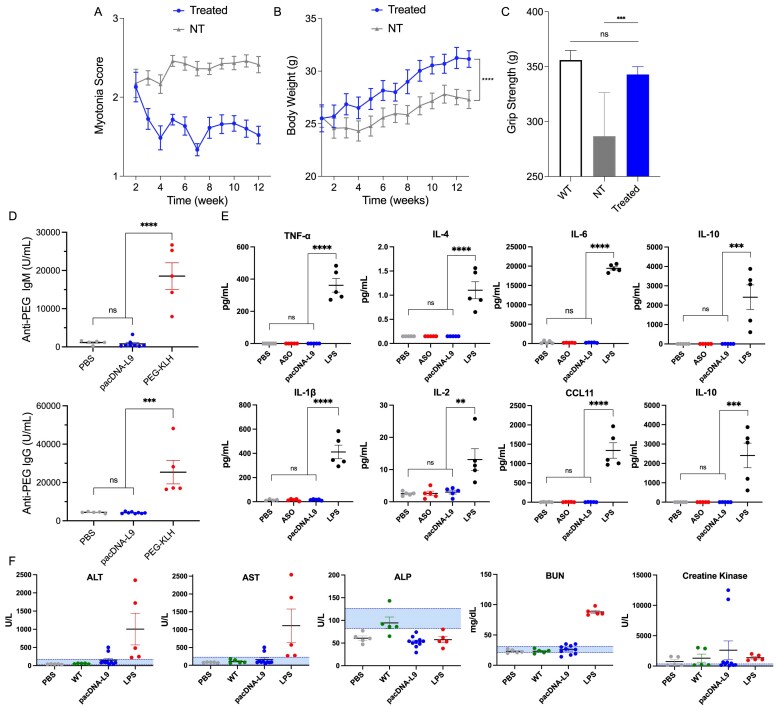
Functional effects of pacDNA-L9 treatment in HSA^LR^ mice and safety evaluation. HSA^LR^ mice received intravenous injections of pacDNA-L9 10.6 mg/kg/day for four consecutive days, followed by once weekly 10.6 mg/kg from weeks 1 to 12, and were monitored compared to nontreated littermates (NT), (*n* = 10/group). (**A**) Myotonia scores measured weekly. The response to each pinch was classified as severe myotonia (>1 s, 3), myotonia (0.5–1 s, 2), quick recovery myotonia (<0.5 s, 1), single leg myotonia (0.5), or no myotonia (0). (**B**) Body weight measurements were recorded throughout the administration period. (**C**) Week 12 grip strength analysis demonstrates improved muscle strength in the treated group compared to the nontreated group. The mean and SEM (*n* = 10 per group) in WT, nontreated HSA^LR^ (NT), and treated 10.6 mg/kg/dose HSALR are displayed (**P < .01, ***P < .001, and ****P < .0001 by one-way ANOVA followed by Tukey’s multiple comparisons correction). (**D**) Anti-PEG IgM and IgG antibody generation evaluated after 16 doses (10.6 mg/kg) of pacDNA-L9 by ELISA (*n* = 8). PBS (*n* = 5) was used as the negative control and PEG-KLH (*n* = 5) as the positive control. (**E**) Cytokine and chemokine levels in HSA^LR^ mice serum 4 h after i.v. injection of PBS, free L9-ASO, pacDNA-L9 (10.6 mg/kg), or LPS (2 mg/kg). (**F**) Liver and kidney function biomarker levels in HSA^LR^ mice serum after i.v. injection of PBS, pacDNA-L9 (10.6 mg/kg), or LPS (2 mg/kg), or in FVB/N WT (WT) mice. The shaded area represents the healthy reference range in FVB/N mice. (**P < .01, ***P < .001, ****P < .0001, one-way ANOVA). Error bars indicate ±SEM.

Next, we evaluated the phenotypic response of the pacDNA-L9 treatment in HSA^LR^ mice. DM1 in humans is characterized by progressive muscle wasting and weakness, alongside myotonia, which refers to the delayed relaxation of muscles after contraction. Myotonia is a hallmark feature in both DM1 patients and the HSA^LR^ mouse model and contributes significantly to physical impairment. HSA^LR^ mice (8 weeks old) were administered i.v. with pacDNA-L9 at 10.6 mg/kg/day for four consecutive days, followed by weekly (10.6 mg/kg/week) dosing from weeks 1 through 12. A blinded hindlimb tail-pinch assay was performed each week to evaluate myotonia (delayed muscle relaxation). Significant improvements in myotonia severity and recovery time were observed starting in the 3rd week of treatment (*P* < .01), with maximal therapeutic effect achieved by the 7th week. Myotonia scores for the treated group remained consistently lower than those for the nontreated group (*P* < .001 from weeks 7 to 12; Fig. 5A). Additionally, mice in the treatment group showed a progressive increase in body weight compared to the nontreated group, with the male group showing a 14.6% increase and the female group showing a 9.1% increase over nontreated mice of the same age at week 12 (Fig. 5B). To further assess the functional impact of pacDNA-L9 treatment, we conducted a forelimb and hindlimb grip strength test to evaluate muscle strength in HSA^LR^ mice. At week 12, the treated mice were significantly stronger than their untreated littermates (*P* < .001), close to WT levels (Fig. 5C). These findings suggest that pacDNA-L9 effectively improves muscle strength and alleviates myotonia following treatment, with sustained efficacy throughout the dosing period.

Macromolecular therapeutics can elicit adaptive immune responses that lead to loss of drug activity for subsequent doses. To measure this effect, anti-drug immunogenicity after 16 doses of pacDNA-L9 was evaluated for anti-PEG IgM and IgG response. In serum samples collected 1 week after the final injection, minimal anti-PEG IgM and IgG antibodies were detected, while the PEG-KLH positive control group showed significant levels of both IgM and IgG (Fig. 5D). To assess acute immune system activation, a panel of cytokines and chemokines was measured using HSA^LR^ mouse serum collected 4 h after the last injection. For positive control, mice were injected with 2 mg/kg of LPS to induce an acute immune response. No significant elevations in cytokine/chemokine levels were seen between pacDNA-L9-treated and nontreated mice, whereas the LPS-treated group exhibited significantly elevated levels in all 16 tested cytokines/chemokines, indicating that pacDNA-L9 does not trigger acute inflammatory responses (Fig. 5E and Supplementary Fig. S12). Liver function markers, including alanine aminotransferase (ALT), alkaline phosphatase (ALP), aspartate aminotransferase (AST), albumin, total bilirubin (TBIL), and total protein (TP), as well as renal function markers, such as blood urea nitrogen (BUN) and creatinine, remained within normal ranges, indicating that pacDNA-L9 treatment does not cause hepatic or renal dysfunction (Fig. 5F and Supplementary Fig. S13). Taken together, pacDNA-L9 shows excellent tolerance in mice, with no evidence of significant immune system activation, toxicity, or anti-drug adaptive immunity throughout 12 weeks of repeated dosing.

## Discussion

The challenge of muscular delivery of oligonucleotides was underscored by the discontinuation of the development of *baliforsen*, the first ASO therapy for DM1, in 2017, citing insufficient drug concentration in muscle after subcutaneous injection [76]. TfR-targeted approaches have recently gained interest for muscle delivery, with several in late-stage clinical trials for DM1, including *del-desiran* and *zeleciment basivarsen*, which showed strong promise. However, the complex antibody/Fab conjugate may present challenges in manufacturing, transport, storage, and handling at the clinic. To counter some of the drawbacks of antibody conjugates, peptide ligands that bind TfR1 or other muscle-specific targets are being developed, although these remain untested in the clinic [56, 57, 77, 78].

A second approach to the delivery problem is the use of polycationic substances such as lipids, polymers, or peptides, which improve per-molecule potency by enhancing cell uptake and endosomal escape [12, 79]. While effective *in vitro* and promising in murine and nonhuman primate models, polycationic substances pose toxicity concerns at some therapeutic doses, due to nonspecific lytic activity, unwanted immune system activation, and organ damage [80, 81]. SRP-5051, a cationic peptide-phosphorodiamidate morpholino oligomer conjugate for the treatment of DMD, was discontinued due to the likelihood of having a narrow therapeutic window [82]. Clinical results of DM1 programs using cell-penetrating peptide conjugates, such as VX-670 and PGN-EDODM1, remain to be seen.

As an oligonucleotide delivery modality, pacDNA is novel in both chemistry and mechanism. It achieves comparable potency as the state-of-the-art DM1 therapies despite utilizing an unoptimized ASO [53, 83, 84]. After i.v. injection, the strong blood retention properties of the pacDNA drive ASO distribution to skeletal/cardiac muscles, yielding muscle concentrations that are 3- to 10-fold higher than mAb conjugates and over 100-fold higher than peptide conjugates in mice [52, 85] (Supplementary Fig. S7). While the improved plasma retention also leads to higher drug levels in the kidney and the liver, muscle selectivity (defined as muscle concentration relative to dose-limiting organ, e.g. kidney) also improved significantly compared to free ASO. The broad distribution profile of pacDNA may be particularly advantageous for multisystem diseases such as DM1, which affect not only skeletal muscle but also the central nervous, gastrointestinal, cardiac, and respiratory systems [86]. Critically, 12 weeks of repeated dosing produced no detectable toxic or immunogenic side effects. Blood biochemistry markers remained within the normal range, and no cytokine response related to innate and adaptive immunity was observed. Further, the treatment did not result in anti-drug antibodies, as measured by ELISA for anti-PEG IgG and IgM. These results are consistent with our previous studies (for non-DM1 targets), showing a clean safety profile and non-immunogenicity in both murine and nonhuman primate models [28, 87]. Unlike antibodies and antibody–drug conjugates, the pacDNA is stable at room temperature for at least 1 month and can be repeatedly lyophilized and reconstituted, providing simplicity in storage, transport, and handling. Owing to strong safety characteristics, substantial muscle bioavailability, and a favorable drug profile, the pacDNA could serve as a viable alternative or complementary approach to current drug delivery modalities such as muscle cell surface receptor-targeting antibodies and peptides [25, 26, 54, 56, 66, 88].

As a first-generation platform, the pacDNA has the potential for iterative optimization via polymer backbone modifications [33], linker chemistries [89], ligand attachment to enhance cell targeting (via peptide or lipid conjugation), nuclear uptake, endosomal escape [90–92], and ASO optimization. This modularity will allow the pacDNA structure to evolve alongside our understanding of what drives potency in a disease-specific context. This study establishes a proof-of-concept for this fundamentally new oligonucleotide delivery modality for the treatment of DM1, with implications for broader development in other neuromuscular diseases including myotonic dystrophy type 2 (DM2), DMD, and facioscapulohumeral muscular dystrophy.

## Supplementary Material

gkag619_Supplemental_File

## Data Availability

All data are available in the main text or the supplementary materials. RNA-seq data have been deposited in GEO (https://www.ncbi.nlm.nih.gov/geo/) and will be publicly available under accession number GSE309640 upon publication.
