# Supplementary material for: Bottlebrush polymer conjugates for enhanced antisense oligonucleotide therapy in myotonic dystrophy type 1
Source: Nucleic Acids Res. 2026 Jun 24;54(12):gkag619. doi: 10.1093/nar/gkag619 (PMC13291609; doi:10.1093/nar/gkag619)
Supplement: gkag619_Supplemental_File [file gkag619_supplemental_file.pdf]

Supplementary Materials for

Bottlebrush Polymer Conjugates for Enhanced Antisense Oligonucleotide Therapy in Myotonic Dystrophy Type 1

Yao Li, Christopher Oetheimer, *et al.*

*Ke Zhang, Corresponding author. Email: k.zhang@northeastern.edu

This PDF file includes:

Supplementary Fig. S1 to S13
Supplementary Table S1 to S3
Abbreviations

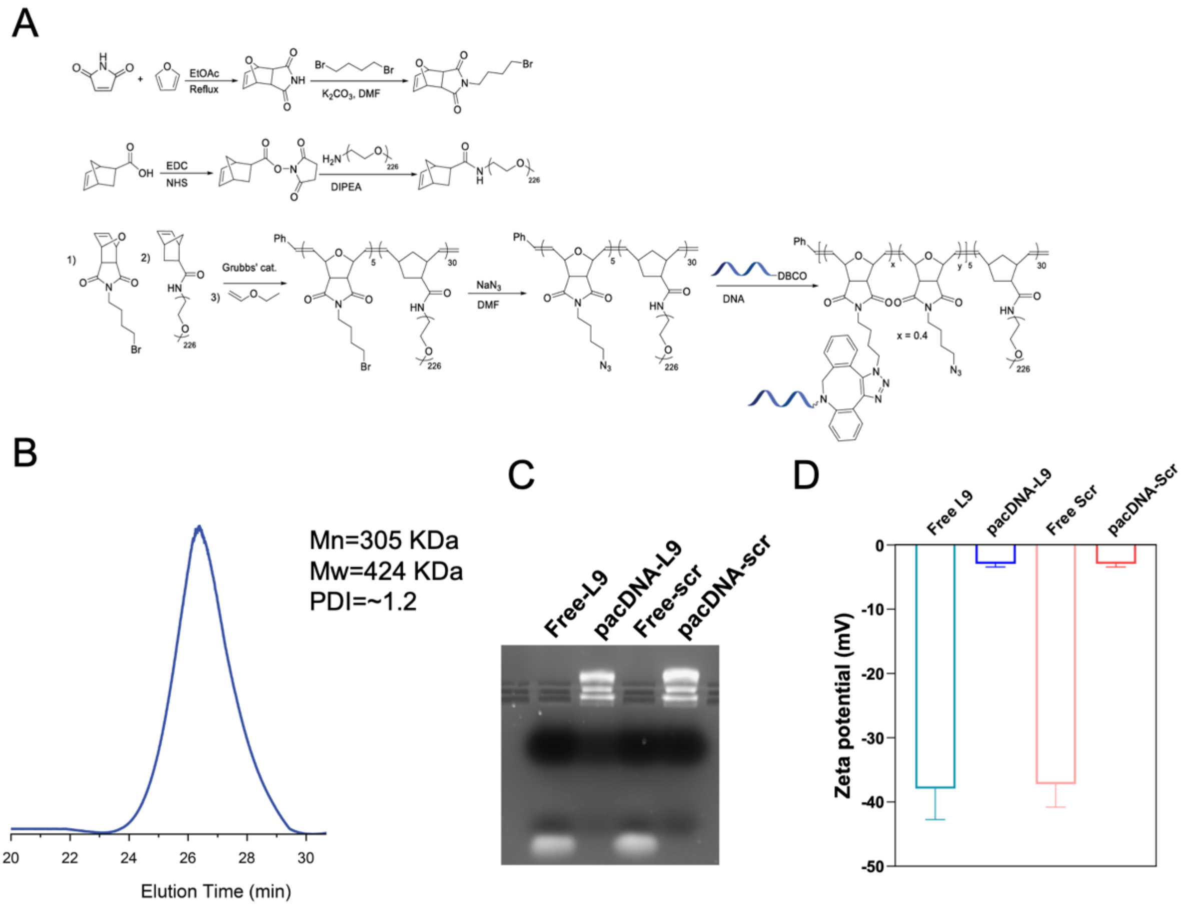

Supplementary Figure S1. (A) Synthesis scheme of the pacDNA conjugate. DBCO-functionalized ASO shown in blue (~2.0 ASO per polymer) **(B)** Aqueous gel permeation chromatograph of the pacDNA-L9 conjugate. Molecular weight (Mn and Mw) and polydispersity (PDI) were approximated by DMF-GPC. **(C)** Agarose gel (2%) electrophoresis separation of free L9 ASO (lane 1), pacDNA-L9 (lane 2), free L9-Scr ASO (lane 3), and pacDNA-Scr (lane 4). **(D)** Zeta potential (ζ) measurements of the pacDNA-L9, pacDNA-Scr, free L9 ASO, and L9-Scr ASO in Nanopure water. Error bars indicate ±s.d.

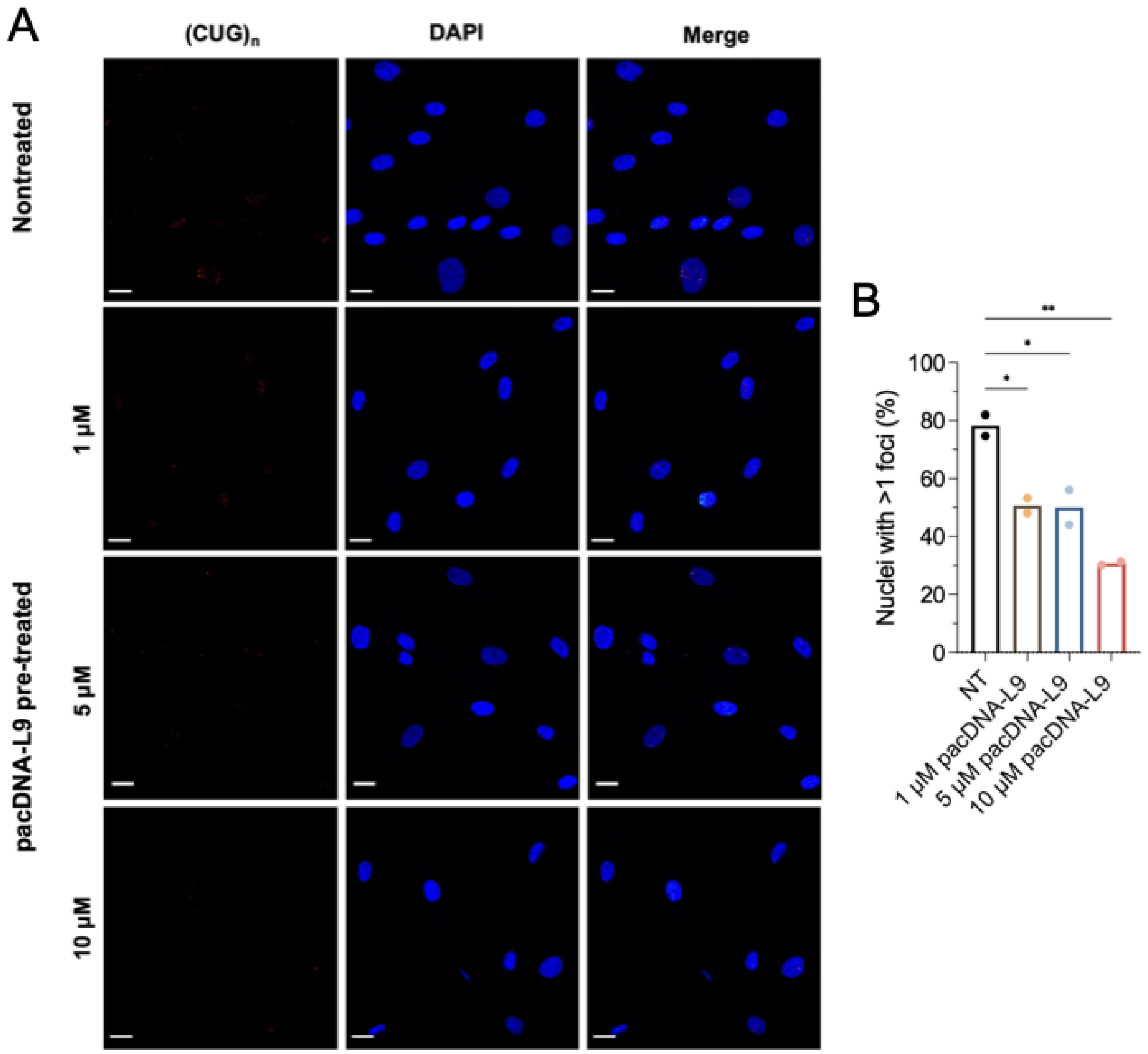

Supplementary Figure S2. (A) Dose-responsive fluorescence *in situ* hybridization experiment of human DM1 fibroblasts (GM03989) pretreated for 24 h with 1 µM, 5 µM, or 10 µM pacDNA-L9. Red: nuclear CUG^{exp} foci; blue: nuclei. Scale bars: 20 µm. **(B)** Quantification of the percentage of nuclei with foci > 1 in nontreated and pacDNA-L9 treated cells (~150 nuclei counted per sample per replicate).

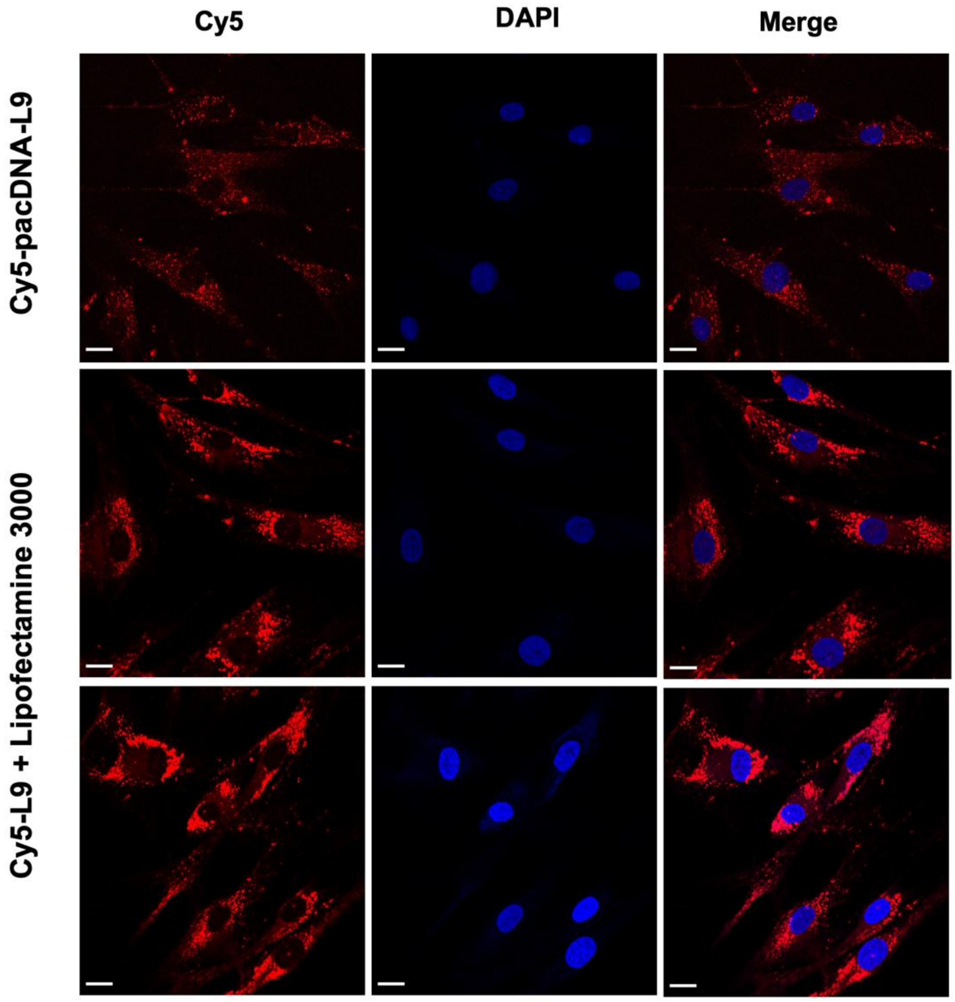

Supplementary Figure S3. DM1 fibroblasts (GM03989) treated with Cy5-labeled pacDNA-L9 (1 µM, ASO basis) or free Cy5-L9 ASO polyplex with Lipofectamine 3000 for 24 h. Red: ASO; blue: nuclei. Scale bars: 20 µm.

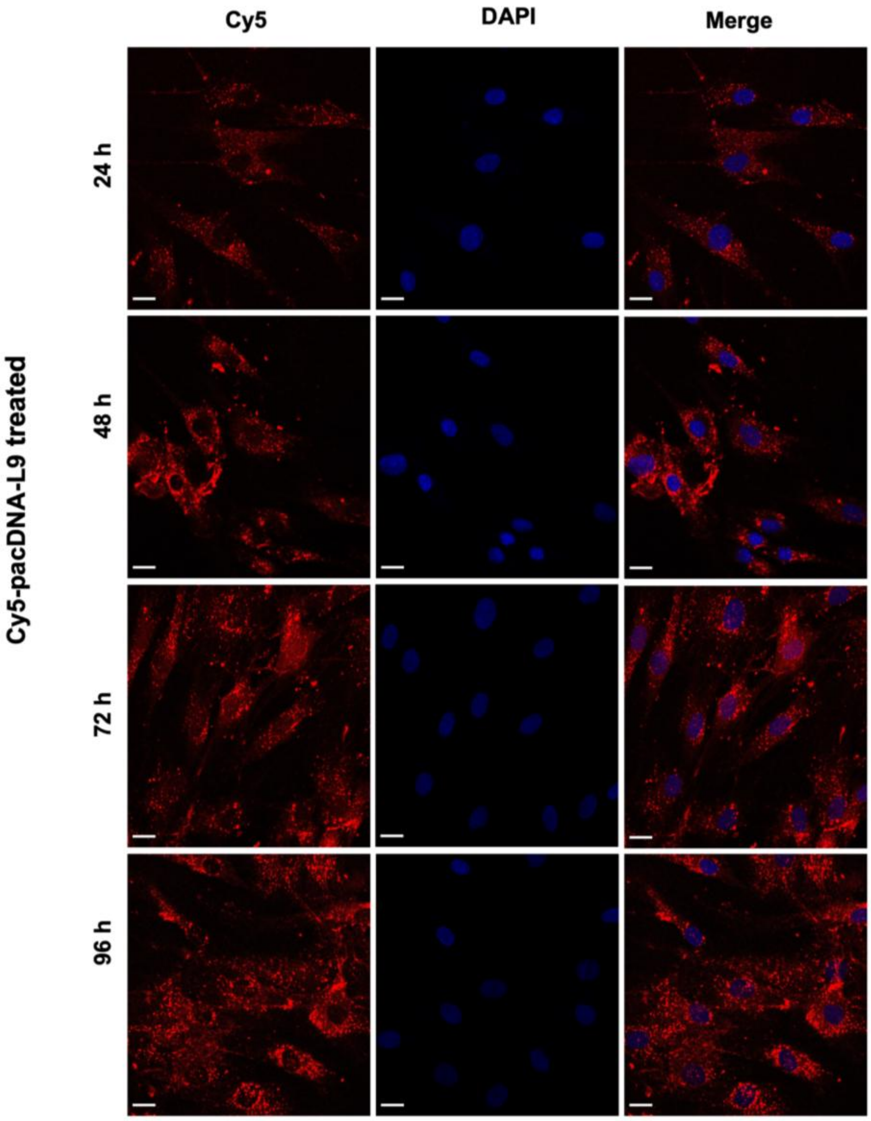

Supplementary Figure S4. Human DM1 fibroblasts (GM03989) treated with 1 µM (ASO basis) Cy5-pacDNA-L9 for 24, 48, 72, and 96 h. Red: pacDNA-L9; blue: nuclei. Scale bars: 20 µm.

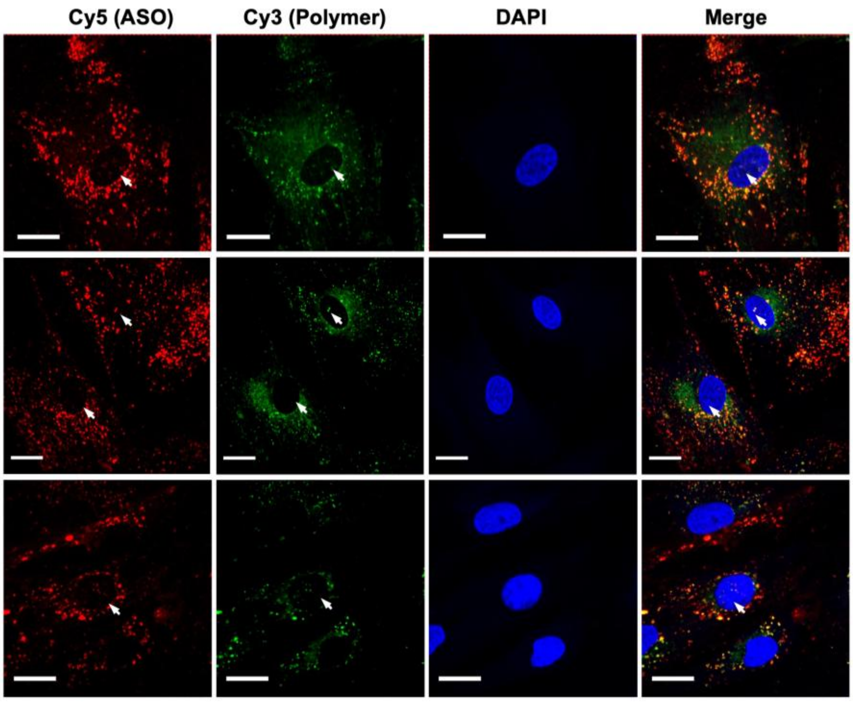

Supplementary Figure S5. Confocal microscopy of human DM1 fibroblasts (GM03989) treated with dual-labeled pacDNA-L9 for 24 h, showing colocalization. Red: Cy5-L9 ASO; green: Cy3-bottlebrush polymer; blue: nuclei. Scale bars: 20 μm.

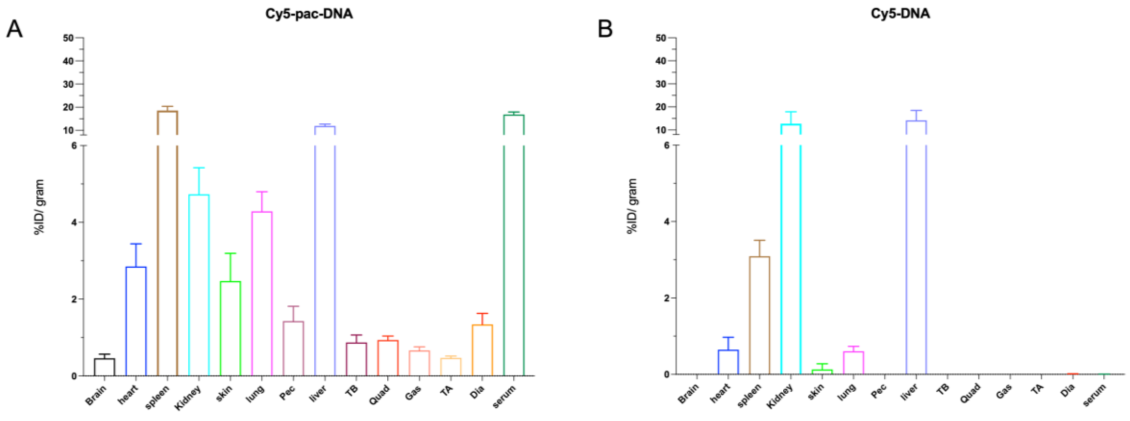

Supplementary Figure S6. Biodistribution of Cy5-labeled pacDNA and free ASO. (A, B) *Ex vivo* biodistribution of Cy5-pacDNA-L9 and Cy5-L9 in various tissues and organs measured over 3 days (n=3) in HSA^{LR} mice. The y axis shows the percent of injected dose per gram of tissue (%ID/g). Quad-quadriceps; Gas-gastrocnemius; TA-tibialis anterior; Pec–pectoralis; Dia–diaphragm.

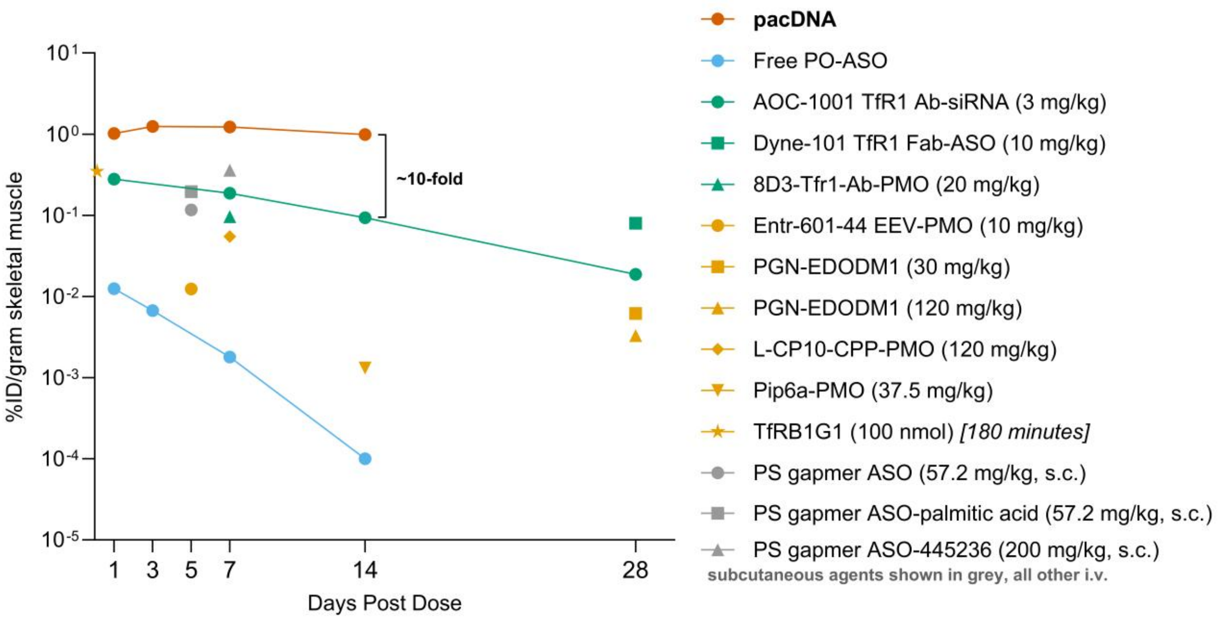

Supplementary Figure S7: Skeletal muscle accumulation (% injected dose/gram muscle) of pacDNA and free ASO (Figure 2 of this manuscript) along with published data from peptide and antibody conjugates in mice (data converted to %ID/g). Green: Tfr1 antibody or Fab conjugates; yellow: peptide conjugates which are either muscle targeting or cationic; grey: free ASO or lipid-conjugated ASOs. The free ASO and lipid-ASO conjugates were dosed s.c.; all other agents were injected i.v. %ID/g values were calculated using published concentrations and individual ASO/siRNA drug molecular weights, assuming mouse bodyweight = 25 g. The x axis represents time since the final dose if multiple doses were administered. Sources: (Weeden, 2025; Geall, 2021; Holland, 2025; Entrada Therapeutics, Inc. 2023 Annual Report to Securityholders; Klein, 2019; Schneider, 2025; Prakash, 2019; Crook, 2020; Hammond, 2022; Wheeler, 2012). Dyne-101: gastrocnemius; Pip6a-PMO: quadriceps; L-CP10-COO-PMO: quadriceps; PGN-EDODM1: skeletal muscle (unspecified); AOC-1001: quadriceps; PS gapmer ASO and PS gapmer ASO-palmitic acid: quadriceps; Entr-601-44: skeletal muscle (unspecified); TfRB1G1: skeletal muscle (unspecified); 8D3-Tfr1-Ab-PMO: quadriceps; ASO-445236: quadriceps.

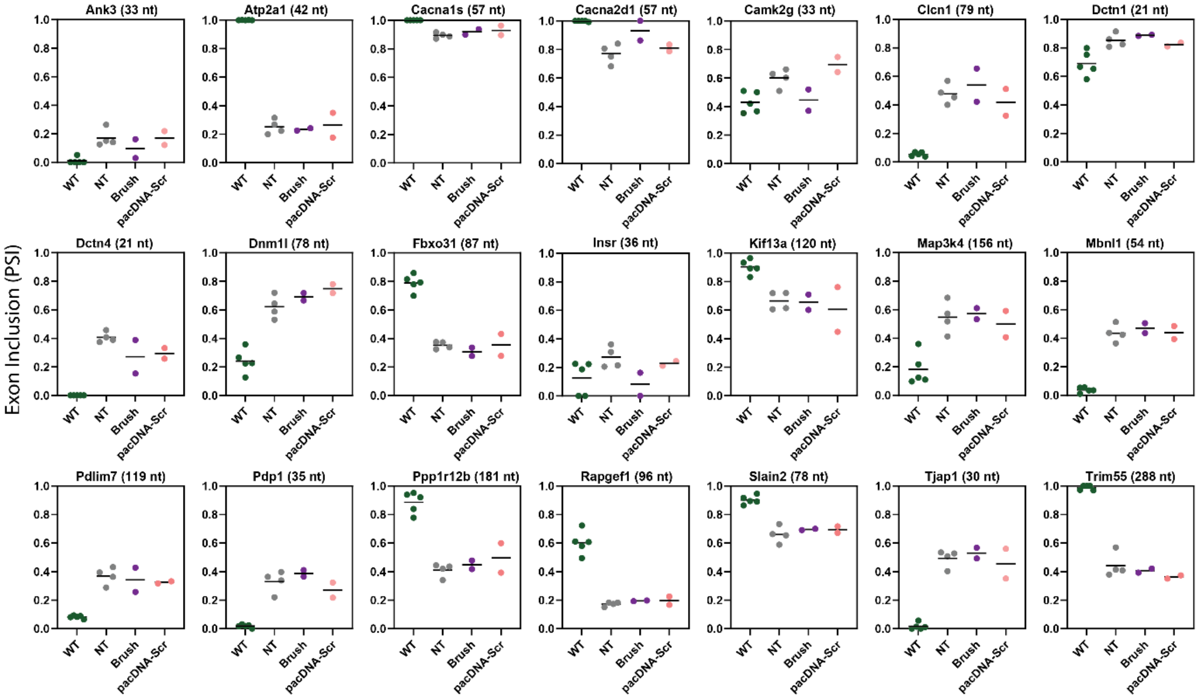

Supplementary Figure S8. Percent spliced in (PSI) of 21 DM1-associated splice events in wildtype (n=5), nontreated HSA^{LR} (n=4), brush (equimolar to pacDNA-L9, n=2) and pacDNA-Scr (5.3 mg/kg, n=2) treated HSA^{LR} quadriceps, two weeks post-injection.

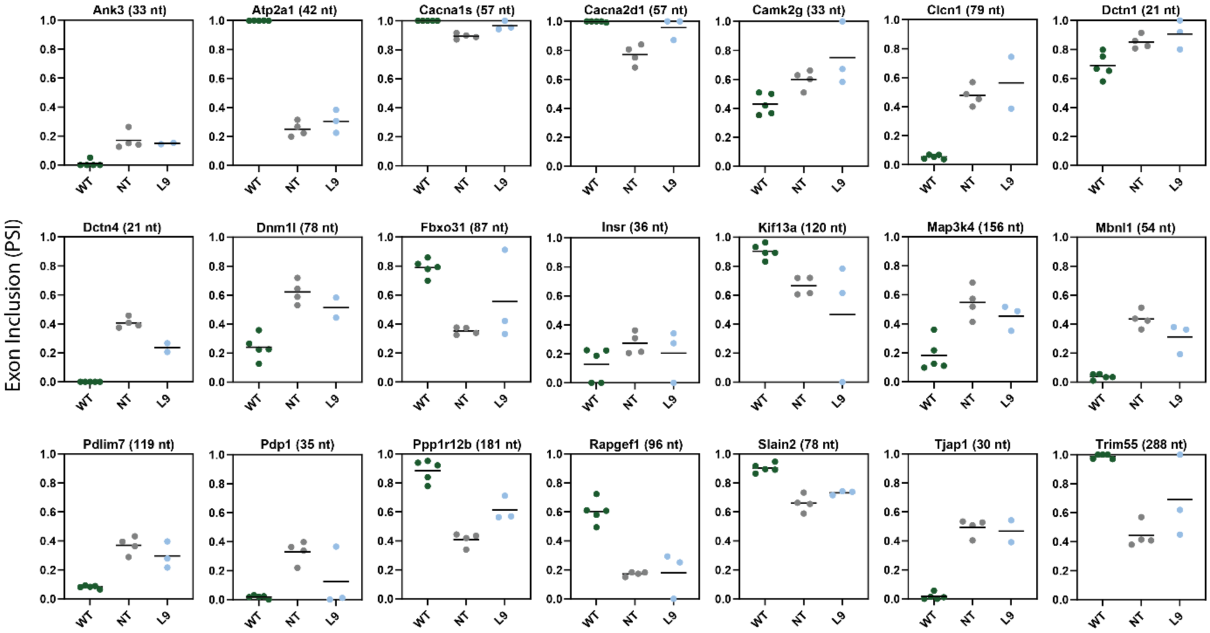

Supplementary Figure S9. Percent spliced-in (PSI) graphs for 21 DM1-associated splice events in wildtype (n=5), nontreated HSA^{LR} (n=4) and free L9 ASO-treated HSA^{LR} mice (5.3 mg/kg, n=3) in quadriceps two weeks post-injection.

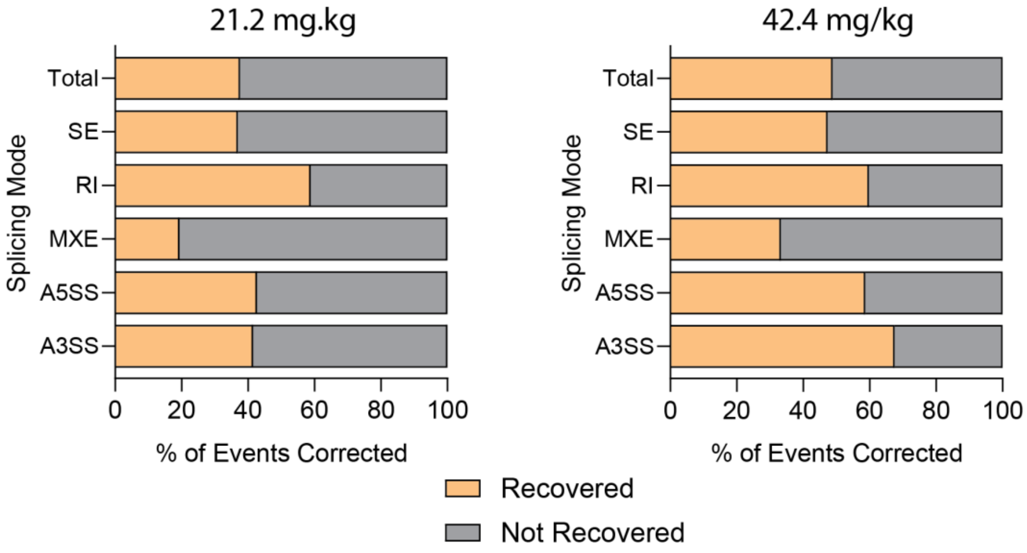

Supplementary Figure S10. Splicing mode correction in 21.2 mg/kg and 42.4 mg/kg cohort. 1287 splicing events comprising alternative three primes splice site (A3SS), alternative five primes splice site (A5SS), mixed exon (MXE), retained intron (RI), and skipped exon (SE) events identified as dysregulated in DM1.

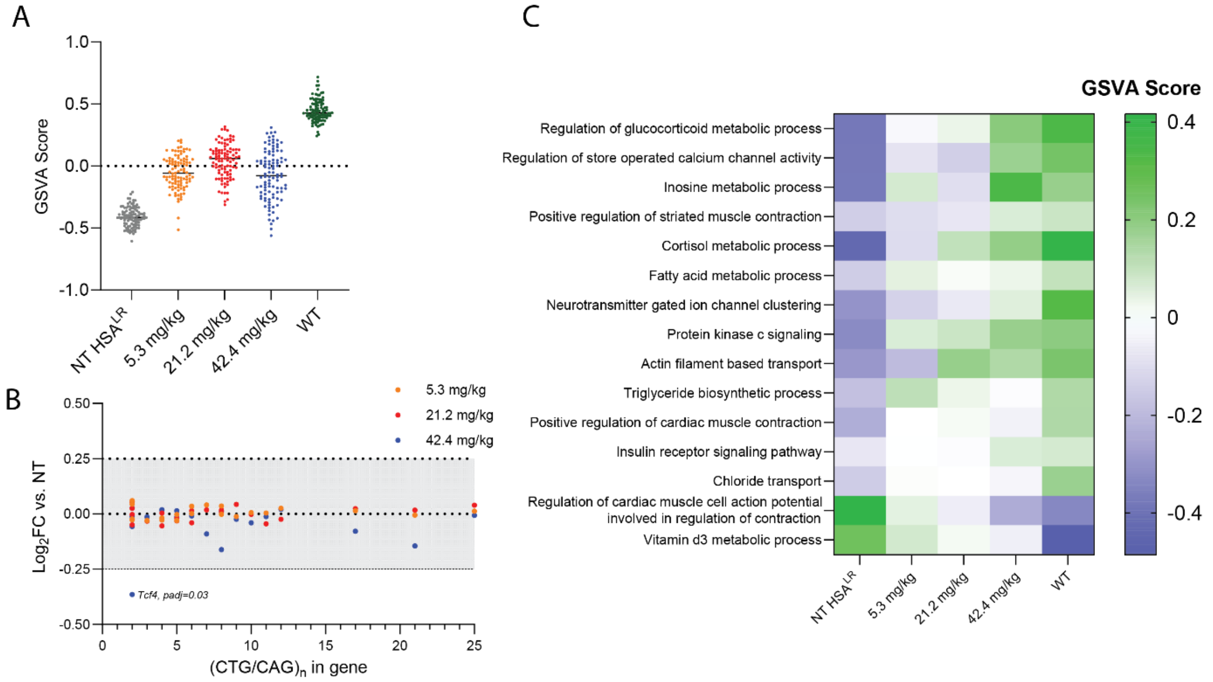

Supplementary Figure S11. (A) Gene set variation analysis (GSVA) scores of the 100 lowest score (most downregulated) GO biological processes in nontreated HSA^{LR} samples compared to wildtype across all treatment groups. **(B)** Select GO biological process GSVA scores visualized by heatmap across all treatment groups. **(C)** Expression levels of 21 endogenous genes containing short $(CUG)_n$ (n=2-25) RNA tracts in pacDNA-L9 treated mice vs. NT (Table S3).

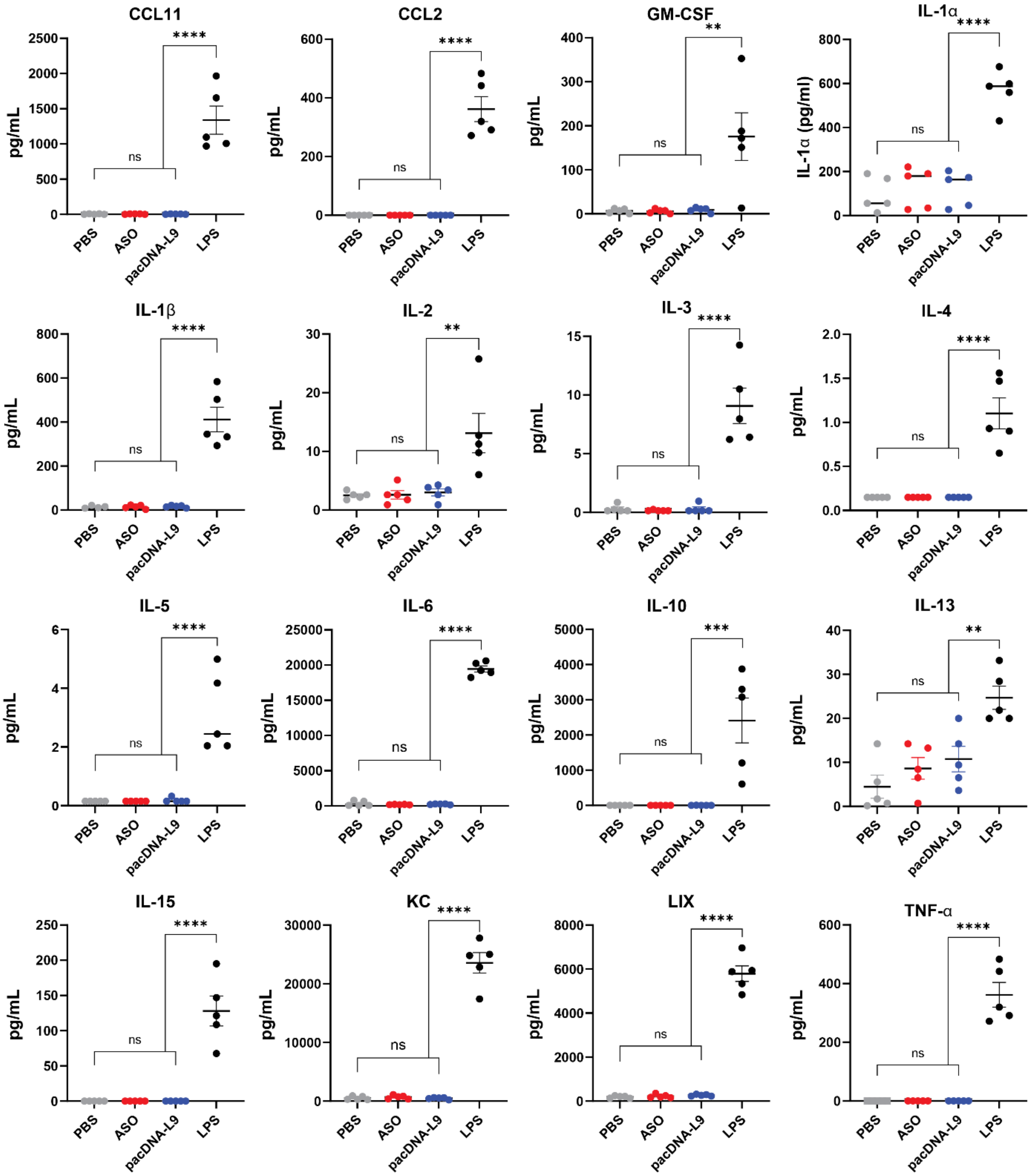

Supplementary Figure S12. Cytokine and chemokine levels in HSA[LR] mice serum 4 h after i.v. injection of PBS, free L9-ASO, pacDNA-L9 (10.6 mg/kg), or LPS (2 mg/kg) (n=5 per group) (****p<0.0001, ***p<0.001, **p<0.01, one-way ANOVA). Error bars indicate ±s.e.m.

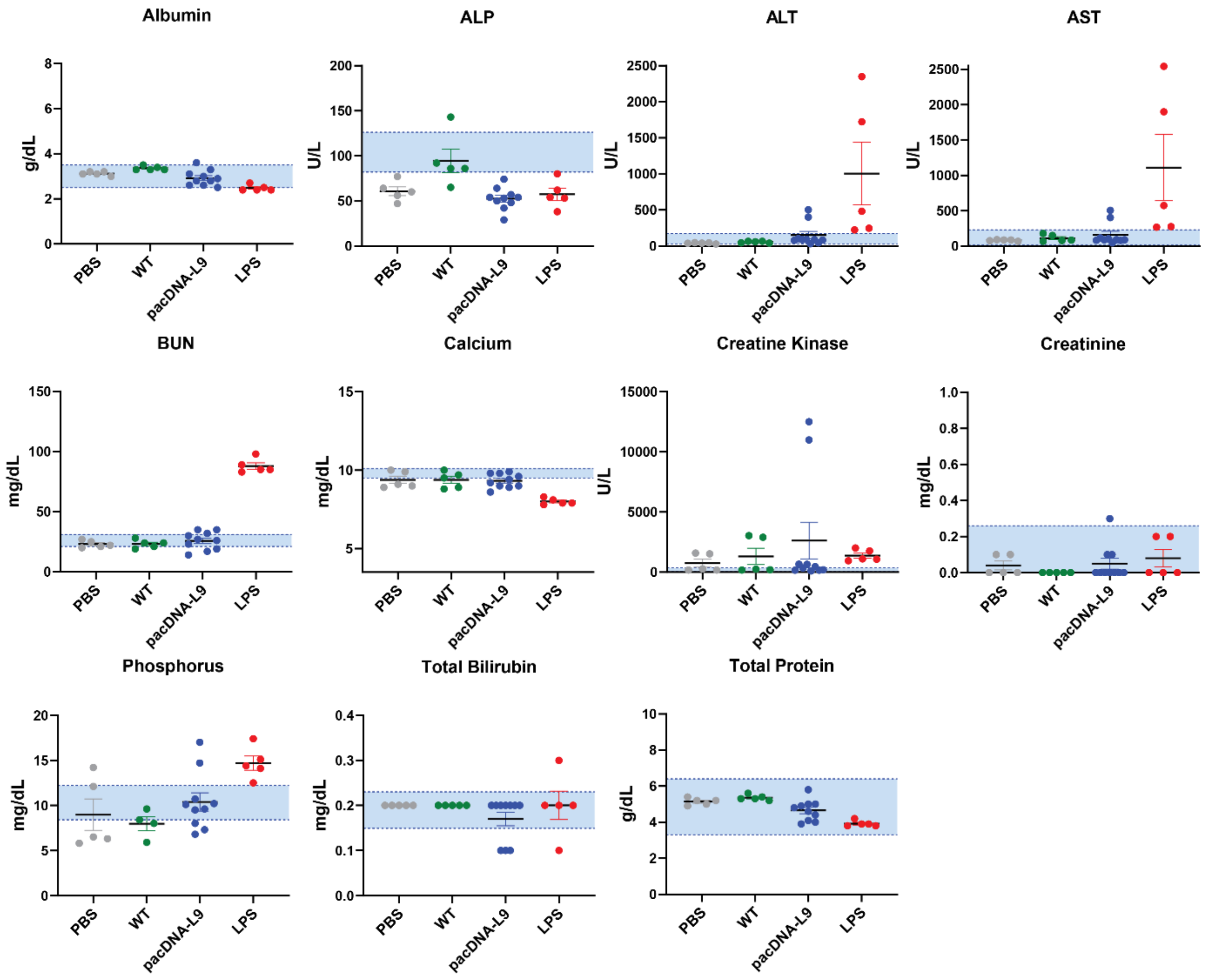

Supplementary Figure S13. Liver function and renal function biomarker levels in HSA^{LR} mice serum collected after i.v. injection of PBS (n=5), pacDNA-L9 (10.6 mg/kg) (n=10), or LPS (2 mg/kg) (n=5), or in FVB/N wildtype (WT) mice (n=5). The blue shaded area represents the healthy reference range in FVB/N mice. Error bars indicate ±s.e.m.

Gene Symbol	ENSMUSG_ID	Gene Symbol	ENSMUSG_ID
Ntf5		Tnfaip8l1	ENSMUSG00000041759
Gm50285		2310039L15Rik	
Mkrn3	ENSMUSG00000049436	Marchf9	ENSMUSG00000041731
Gm11382		Itih4	ENSMUSG00000026639
Fosb	ENSMUSG00000013205	Cd68	ENSMUSG00000018774
Pglyrp1	ENSMUSG00000050822	Mmp3	ENSMUSG00000043613
Hes1	ENSMUSG00000022528	Il6ra	ENSMUSG00000027947
Epb41l5	ENSMUSG00000025810	Gm34220	
Zscan18	ENSMUSG00000056749	Gm34804	
Gm49454		Gm44717	
Gm16159		Serpina3n	ENSMUSG00000021060
Gas2l3	ENSMUSG00000035295	Lgals3	ENSMUSG00000027797
Pthlh	ENSMUSG00000021998	Crip3	ENSMUSG00000033813
Tas1r1	ENSMUSG00000031673	ENSMUSG00000120664	ENSMUSG00000120664
Mir6338		Lcn2	ENSMUSG00000026822
Gm44899		1700102H20Rik	
Spns3	ENSMUSG00000046179	Camk2n2	ENSMUSG00000032115
Nfatc2	ENSMUSG00000025542	Ntng2	ENSMUSG00000056647
Socs3	ENSMUSG00000013729	Rnf212b	ENSMUSG00000073708
Hmga2-ps1		Rrad	ENSMUSG00000032550
Rhbdl3	ENSMUSG00000037012	Mfap4	ENSMUSG00000029461
C330018A13Rik		Tppp3	ENSMUSG00000031834
5930438M14Rik		ENSMUSG00000120176	ENSMUSG00000120176
Ildr1	ENSMUSG00000059401	Serpine1	ENSMUSG00000037411
Creg2	ENSMUSG00000014599	Icam1	ENSMUSG00000037405
Gm50194		Cacnb4	ENSMUSG00000032097
Ptx3	ENSMUSG00000027832	Cfap77	ENSMUSG00000052504
1700110C19Rik		Clca1	ENSMUSG00000062627
Fos	ENSMUSG00000021250	Gm10433	
Nrg2	ENSMUSG00000056644	Cilp	ENSMUSG00000037336
Actr3b	ENSMUSG00000027790	Gm28040	
Gm6665		Atf3	ENSMUSG00000026628
Egr1	ENSMUSG00000038418	S100a4	ENSMUSG00000023341
Tppp2	ENSMUSG00000034428	Ncmap	ENSMUSG00000050295
Cntnap2	ENSMUSG00000061915	Ncaph	ENSMUSG00000031537
1010001B22Rik		Xirp1	ENSMUSG00000032446
Klhl6	ENSMUSG00000030093	Tiam2	ENSMUSG00000048095
Adamts9	ENSMUSG00000058880	Prg4	ENSMUSG00000028152
Lgi2	ENSMUSG00000045321	Cacna1i	ENSMUSG00000021897
Cnga3	ENSMUSG00000032150	Gm40634	
Gm11266		Gm11827	
Dok5	ENSMUSG00000040152	4930556M19Rik	
Cdcp1	ENSMUSG00000020950	Slit1	ENSMUSG00000031558
Gm15132		Hspb1	ENSMUSG00000004951
Gask1a	ENSMUSG00000066687	Batf2	ENSMUSG00000059019
BC023105		Ankrd1	ENSMUSG00000002007
Tesmin	ENSMUSG00000032503	Irf5	ENSMUSG00000029770

E2f7	ENSMUSG00000056478	AW046200	
Gm44193		Lmod2	ENSMUSG00000032549
Dhcr24	ENSMUSG00000019224	Arhgap33	ENSMUSG00000046427
Uhrf1	ENSMUSG00000040204	2310039L15Rik	
Olfr93	ENSMUSG00000062227	Marchf9	ENSMUSG00000041731
Tmem158	ENSMUSG00000027199	Itih4	ENSMUSG00000026639
Prepl	ENSMUSG00000050069	Cd68	ENSMUSG00000018774
Gm20758		Mmp3	ENSMUSG00000043613
Mss51	ENSMUSG00000038998	Il6ra	ENSMUSG00000027947
Ttc7	ENSMUSG00000030471	Gm34220	
Cracd	ENSMUSG00000052837	Gm34804	
Irx3os		Gm44717	
Rhpn2	ENSMUSG00000041146	Serpina3n	ENSMUSG00000021060
Cdhr4	ENSMUSG00000050765	Lgals3	ENSMUSG00000027797
Gm45163		Crip3	ENSMUSG00000033813
Gm53023		ENSMUSG00000120664	ENSMUSG00000120664
Amy1	ENSMUSG00000074694	Lcn2	ENSMUSG00000026822
Gm36325		1700102H20Rik	
2310068J16Rik		Camk2n2	ENSMUSG00000032115
Gadd45b	ENSMUSG00000015312	Ntng2	ENSMUSG00000056647
2900093K20Rik		Rnf212b	ENSMUSG00000073708
Mettl21c	ENSMUSG00000036855	Rrad	ENSMUSG00000032550
Col9a1	ENSMUSG00000026147	Mfap4	ENSMUSG00000029461
Spata6l	ENSMUSG00000061088	Tppp3	ENSMUSG00000031834
Meg3	ENSMUSG00000021268	ENSMUSG00000120176	ENSMUSG00000120176
ENSMUSG00000120806	ENSMUSG00000120806	Serpine1	ENSMUSG00000037411
Gm4107		Icam1	ENSMUSG00000037405
6720468P15Rik		Cacnb4	ENSMUSG00000032097
Esyt3	ENSMUSG00000020911	Cfap77	ENSMUSG00000052504
Eif4e1b	ENSMUSG00000063659	Clca1	ENSMUSG00000062627
Gm31579		Gm10433	
Amhr2	ENSMUSG00000029402	Cilp	ENSMUSG00000037336
Spata33	ENSMUSG00000074411	Gm28040	
Atp1b4	ENSMUSG00000062939	Atf3	ENSMUSG00000026628
Dnase2b	ENSMUSG00000041607	S100a4	ENSMUSG00000023341
Ttc39a	ENSMUSG00000032402	Ncmap	ENSMUSG00000050295
Cstad	ENSMUSG00000057880	Ncaph	ENSMUSG00000031537
Rapgef4os3		Xirp1	ENSMUSG00000032446
Gm17218		Tiam2	ENSMUSG00000048095
Neu2	ENSMUSG00000039584	Prg4	ENSMUSG00000028152
2310015K22Rik		Cacna1i	ENSMUSG00000021897
Hcn2	ENSMUSG00000053058	Gm40634	
Btbd8	ENSMUSG00000020696	Gm11827	
Wnt5b	ENSMUSG00000036856	4930556M19Rik	
Plekhd1	ENSMUSG00000020473	Slit1	ENSMUSG00000031558
Odf3l2	ENSMUSG00000052357	Hspb1	ENSMUSG00000004951
Cdk1	ENSMUSG00000019942	Batf2	ENSMUSG00000059019
Fam81b	ENSMUSG00000003348	Ankrd1	ENSMUSG00000002007

6430548M08Rik		Irf5	ENSMUSG00000029770
Th	ENSMUSG00000000701	AW046200	
Irx3	ENSMUSG00000031734	Lmod2	ENSMUSG00000032549
ENSMUSG00000120730	ENSMUSG00000120730	Arhgap33	ENSMUSG00000046427
Tnfaip8l1	ENSMUSG00000041759	Hhipl1	ENSMUSG00000047910
2310039L15Rik		Madd	ENSMUSG00000026556
Marchf9	ENSMUSG00000041731	Ppm1e	ENSMUSG00000002083
Itih4	ENSMUSG00000026639	Calml4	ENSMUSG00000047139
Cd68	ENSMUSG00000018774	4930481A15Rik	
Mmp3	ENSMUSG00000043613	Gm26782	
Il6ra	ENSMUSG00000027947	Tnfrsf12a	ENSMUSG00000023906
Gm34220		Lamc2	ENSMUSG00000026479
Gm34804		4930556M19Rik	
Gm44717		Slit1	ENSMUSG00000031558
Serpina3n	ENSMUSG00000021060	Hspb1	ENSMUSG00000004951
Lgals3	ENSMUSG00000027797	Batf2	ENSMUSG00000059019
Crip3	ENSMUSG00000033813	Ankrd1	ENSMUSG00000002007
ENSMUSG00000120664	ENSMUSG00000120664	Irf5	ENSMUSG00000029770
Lcn2	ENSMUSG00000026822	AW046200	
1700102H20Rik		Lmod2	ENSMUSG00000032549
Camk2n2	ENSMUSG00000032115	Arhgap33	ENSMUSG00000046427
Ntng2	ENSMUSG00000056647	Hhipl1	ENSMUSG00000047910
Rnf212b	ENSMUSG00000073708	Madd	ENSMUSG00000026556
Rrad	ENSMUSG00000032550	Ppm1e	ENSMUSG00000002083
Mfap4	ENSMUSG00000029461	Calml4	ENSMUSG00000047139
Tppp3	ENSMUSG00000031834	4930481A15Rik	
ENSMUSG00000120176	ENSMUSG00000120176	Gm26782	
Serpine1	ENSMUSG00000037411	Tnfrsf12a	ENSMUSG00000023906
Icam1	ENSMUSG00000037405	Lamc2	ENSMUSG00000026479
Cacnb4	ENSMUSG00000032097	Prg4	ENSMUSG00000028152
Cfap77	ENSMUSG00000052504	Cacna1i	ENSMUSG00000021897
Clca1	ENSMUSG00000062627	Gm40634	
Gm10433		Gm11827	
Cilp	ENSMUSG00000037336		
Gm28040			
Atf3	ENSMUSG00000026628		
S100a4	ENSMUSG00000023341		
Ncmap	ENSMUSG00000050295		
Ncaph	ENSMUSG00000031537		
Xirp1	ENSMUSG00000032446		
Tiam2	ENSMUSG00000048095		

Supplementary Table S1. Gene symbols and ENSMUSG IDs in Figure 4D heatmap.

Exon_ID	Gene Name	Gene_ID	Chromosome
ENSMUSE00000360150	Hes1	ENSMUSG00000022528	chr16
ENSMUSE00001406312	Eef2	ENSMUSG00000034994	chr10
ENSMUSE00001462927	Unknown	Unknown	chrNA
ENSMUSE00000476481	Socs3	ENSMUSG00000053113	chr11
ENSMUSE00000674408	Kcna7	ENSMUSG00000038201	chr7
ENSMUSE00001491596	Hmga2-ps1	ENSMUSG00000121135	chr1
ENSMUSE00001295963	Prkg1	ENSMUSG00000052920	chr19
ENSMUSE00000366333	Npc2	ENSMUSG00000021242	chr12
ENSMUSE00000796977	Gpt2	ENSMUSG00000031700	chr8
ENSMUSE00000770571	Spns3	ENSMUSG00000020798	chr11
ENSMUSE00000551022	Actr3b	ENSMUSG00000056367	chr5
ENSMUSE00001394300	Rpl4	ENSMUSG00000032399	chr9
ENSMUSE00000358925	Pygb	ENSMUSG00000033059	chr2
ENSMUSE00000444047	Prepl	ENSMUSG00000024127	chr17
ENSMUSE00000530732	Synm	ENSMUSG00000030554	chr7
ENSMUSE00000121140	Mss51	ENSMUSG00000021815	chr14
ENSMUSE00000811913	Des	ENSMUSG00000026208	chr1
ENSMUSE00000764115	Mrpl53	ENSMUSG00000030037	chr6
ENSMUSE00000135592	Map3k4	ENSMUSG00000014426	chr17
ENSMUSE00000108712	Cacng1	ENSMUSG00000020722	chr11
ENSMUSE00000739070	Atp1a2	ENSMUSG00000007097	chr1
ENSMUSE00000907794	Gm6665	ENSMUSG00000091561	chr18
ENSMUSE00000150608	Dhcr24	ENSMUSG00000034926	chr4
ENSMUSE00000121136	Mss51	ENSMUSG00000021815	chr14
ENSMUSE00001105014	Rian	ENSMUSG00000097451	chr12
ENSMUSE00000121135	Mss51	ENSMUSG00000021815	chr14
ENSMUSE00000183754	Tas1r1	ENSMUSG00000028950	chr4
ENSMUSE00000508157	Dhcr24	ENSMUSG00000034926	chr4
ENSMUSE00000673864	Ptpn3	ENSMUSG00000038764	chr4
ENSMUSE00000818731	Irx3os	ENSMUSG00000046413	chr8
ENSMUSE00000138969	C3	ENSMUSG00000024164	chr17
ENSMUSE00000681760	Ntrk2	ENSMUSG00000055254	chr13
ENSMUSE00000121139	Mss51	ENSMUSG00000021815	chr14
ENSMUSE00001486783	Gm16124	ENSMUSG00000086914	chr9
ENSMUSE00001486783	Gm16124	ENSMUSG00000086914	chr9
ENSMUSE00001159054	Rian	ENSMUSG00000097451	chr12
ENSMUSE00000517786	BC023105	ENSMUSG00000063388	chr18
ENSMUSE00000673862	Ptpn3	ENSMUSG00000038764	chr4
ENSMUSE00001464259	Vwa3b	ENSMUSG00000050122	chr1
ENSMUSE00000121137	Mss51	ENSMUSG00000021815	chr14
ENSMUSE00001421257	Fktn	ENSMUSG00000028414	chr4
ENSMUSE00000662814	Cstad	ENSMUSG00000047363	chr2
ENSMUSE00000121138	Mss51	ENSMUSG00000021815	chr14
ENSMUSE00000121134	Mss51	ENSMUSG00000021815	chr14
ENSMUSE00000118021	Fbp2	ENSMUSG00000021456	chr13
ENSMUSE00001465072	Unknown	Unknown	chrNA
ENSMUSE00001487306	Unknown	Unknown	chrNA
ENSMUSE00000150485	Myo1a	ENSMUSG00000025401	chr10

ENSMUSE00000673856	Ptpn3	ENSMUSG00000038764	chr4
ENSMUSE00000200406	Ctsc	ENSMUSG00000030560	chr7
ENSMUSE00000386019	Nek6	ENSMUSG00000026749	chr2
ENSMUSE00001457082	Timm21	ENSMUSG00000024645	chr18
ENSMUSE00001368633	Gm31579	ENSMUSG00000107488	chr6
ENSMUSE00000642574	Ubtf	ENSMUSG00000020923	chr11
ENSMUSE00000227709	Rrad	ENSMUSG00000031880	chr8
ENSMUSE00000150492	Myo1a	ENSMUSG00000025401	chr10
ENSMUSE00000304093	Gadd45b	ENSMUSG00000015312	chr10
ENSMUSE00000207557	Itm2a	ENSMUSG00000031239	chrX
ENSMUSE00000418634	Tmem158	ENSMUSG00000054871	chr9
ENSMUSE00000352487	Camk2n2	ENSMUSG00000051146	chr16
ENSMUSE00000363743	Fam124b	ENSMUSG00000043230	chr1
ENSMUSE00000393249	Tdrp	ENSMUSG00000050052	chr8
ENSMUSE00000317344	Hlx	ENSMUSG00000039377	chr1
ENSMUSE00001081441	Fbp2	ENSMUSG00000021456	chr13
ENSMUSE00001489672	Gm3235	ENSMUSG00000090778	chr8
ENSMUSE00000110355	Nxn	ENSMUSG00000020844	chr11
ENSMUSE00001342858	Gm35048	ENSMUSG00000103292	chr1
ENSMUSE00000734122	Meg3	ENSMUSG00000021268	chr12
ENSMUSE00000322715	Cacng1	ENSMUSG00000020722	chr11
ENSMUSE00000150480	Myo1a	ENSMUSG00000025401	chr10
ENSMUSE00000673852	Ptpn3	ENSMUSG00000038764	chr4
ENSMUSE00000358523	Plekho1	ENSMUSG00000015745	chr3
ENSMUSE00000527204	Ptpn3	ENSMUSG00000038764	chr4
ENSMUSE00000869993	Cacna1i	ENSMUSG00000022416	chr15

Supplementary Table S2. Gene symbols and ENSMUSE IDs in Figure 4F heatmap.

Gene	$(CTG)_n$/ *$(CAG)_n$	Log_2FC pacDNA-L9 5.3mg/kg vs. NT	Log_2FC pacDNA-L9 21.2mg/kg vs. NT	Log_2FC pacDNA-L9 42.4mg/kg vs. NT	padj pacDNA-L9 5.3mg/kg vs. NT	padj pacDNA-L9 21.2mg/kg vs. NT	padj pacDNA-L9 42.4mg/kg vs. NT
Papss2	2	0.044	0.025	0.026	0.74	0.858	0.855
Bpgm	2	-0.027	-0.05	-0.01	0.915	0.782	0.918
Dmpk	2	-0.023	-0.003	-0.057	0.922	0.989	0.618
Tcf4	2	0.06	0.055	-0.365	0.728	0.748	0.0341
Ltbp3	3	-0.03	-0.033	-0.014	0.86	0.811	0.929
Rpl14	4	-0.028	0.004	0.019	0.903	0.985	0.851
Bri3bp	4	-0.019	-0.054	0.017	0.928	0.648	0.911
Map3k4	5	-0.03	-0.021	-0.015	0.896	0.91	0.895
Notch4	5	-0.003	-0.032	0.014	0.985	0.773	0.947
Ptbp1	6	0.001	-0.04	-0.009	0.998	0.776	0.959
Sdc3	6	0.035	0.014	0	0.856	0.946	1
Armcx6	7	0.04	0.018	-0.091	0.626	0.852	0.594
Mllt3	8	-0.002	0.007	-0.162	0.995	0.977	0.247
Tacc1	8	0.036	0.017	0.012	0.846	0.921	0.934
Txlnb	9	-0.012	0.043	-0.024	0.96	0.77	0.856
Tnfrsf22	10	0.006	-0.002	-0.04	0.971	0.985	0.834
Pcolce	12	0.025	-0.024	0.021	0.854	0.837	0.909
Fgd4	21	-0.006	0.017	-0.145	0.981	0.917	0.336
Mapkap1	25	0.012	0.039	-0.007	0.972	0.844	0.926
Nr3c1*	17	0.014	0.023	-0.079	0.945	0.859	0.583
Dap*	11	0.004	-0.045	-0.012	0.985	0.697	0.95

Supplementary Table S3. Expression levels of endogenous genes containing short (2-25) CTG repeats relative to non-treated (NT) HSA^{LR} mice.

Abbreviations:

3'-UTR: Three prime untranslated region
ASO: Antisense oligonucleotide
CLCN1: Chloride voltage-gated channel 1
CPG: Controlled-pore glass
CUG^{exp}: CUG-repeat expanded RNA
DBCO: Dibenzocyclooctyne
DFO: Desferrioxamine
DLS: Dynamic light scattering
DM1: Myotonic dystrophy type 1
DM2: Myotonic dystrophy type 2
DMD: Duchenne muscular dystrophy
DMPK: DM1 Protein Kinase
DMT: Dimethoxytrityl
DIPEA: *N*,*N*-diisopropylethylamine
EDCI: 1-ethyl-3-(3-dimethylaminopropyl)carbodiimide
ELISA: Enzyme-linked immunosorbent assay
FDR: False discovery rate
FISH: Fluorescence *in situ* hybridization
FSHD: Facioscapulohumeral muscular dystrophy
GPC: Gel permeation chromatography
GSEA: Gene set enrichment analysis
GSVA: Gene set variation analysis
I.V.: Intravenous
%ID/g: Percent injected dose per gram
KLH: Keyhole limpet haemocyanin
LNA: Locked nucleic acid
LPS: Lipopolysaccharide
MBNL1: Muscleblind-like one
pacDNA: Polymer-augmented conjugates of DNA
PEG: Polyethylene glycol
PMO: Phosphorodiamidate morpholino oligomer
PS: Phosphorothioate
PSI: Percent spliced in
rMATS: R multivariate analysis of transcript splicing
RP-HPLC: Reversed-phase high-performance liquid chromatography
SMA: Spinal muscular atrophy
Tfr1: Transferrin receptor 1
^{89}Zr: Zirconium-89
